# A Multi-Responsive Bismuth-Based Hybrid Exhibiting Dual Thermochromism, SHG Activity, and Dielectric Switching Induced by Sequential Phase Transitions

**DOI:** 10.1021/acs.inorgchem.6c02522

**Published:** 2026-07-16

**Authors:** Magdalena N. Rowińska, Oleksandr Korolevych, Tamara J. Bednarchuk, Jan K. Zaręba, Anna Piecha-Bisiorek, Anna Gagor

**Affiliations:** † Institute of Low Temperature and Structure Research, 215275 Polish Academy of Sciences, Okólna 2, Wrocław 50−422, Poland; ‡ Institute of Advanced Materials, Wrocław University of Science and Technology, Wrocław 50−370, Poland; § Faculty of Chemistry, University of Wrocław, F. Joliot−Curie 14, Wrocław 50−383, Poland

## Abstract

Hybrid organic–inorganic halides combining heavy p-block metals and flexible organic cations emerging as versatile frameworks for multifunctional stimuli-responsive materials. Here, we report a simple bismuth-based iodide hybrid, **(But)**_**3**_ **[BiI**_**6**_ **]**, that exhibits coupled thermochromic, dielectric, and nonlinear optical responses governed by sequential temperature-induced phase transitions. Single-crystal X-ray diffraction reveals three orthorhombic polymorphs (*Pnnm*, *Pccn*, and *P*2_1_2_1_2), stabilized through two reversible phase transitions at 260/274 K and 130/150 K upon cooling/heating. The structural evolution is driven by the interplay between discrete BiI_6_ octahedra and highly flexible butylammonium cations interconnected via N–H···I hydrogen bonding and I···I interactions. Temperature-dependent optical measurements reveal pronounced and reversible thermochromism (orange → green → orange), which is well reproduced by density of states calculations, confirming the electronic origin of the color modulation. Broadband dielectric spectroscopy uncovers strong dipolar dynamics in the high-temperature phase and a sharp dielectric switching behavior associated with the first phase transition, while low-temperature ordering leads to a nearly frequency-independent dielectric response. Second-harmonic generation measurements further demonstrate the emergence of a noncentrosymmetric phase below 130 K, enabling nonlinear optical activity. The coexistence of thermochromism, dielectric switching, and switchable symmetry breaking establishes **(But)**_**3**_ **[BiI**_**6**_ **]** as a lead-free multifunctional hybrid, highlighting a viable strategy for designing environmentally benign materials with coupled optical, electrical, and nonlinear optical functionalities for future optoelectronic and photonic applications.

## Graphical abstract

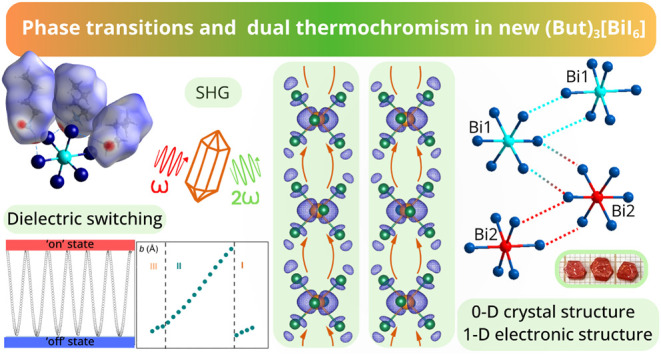

## Introduction

Hybrid organic–inorganic halides have rapidly emerged as one of the most active areas of materials research, largely driven by the exceptional optoelectronic performance of lead-based iodides with perovskite and perovskite-like structures.^1–3^ The defect-tolerant electronic properties and solution-processable nature of these materials have enabled major advances in light-harvesting technologies; however, their chemical instability and lead content remain key barriers to widespread application.^4^ This has prompted an intensive search for lead-free alternatives incorporating metals with stereochemically active lone pairs – most notably Ge­(II), Sn­(II), Bi­(III), and Sb­(III) – that can retain the desirable properties of Pb-based systems while offering improved stability and reduced toxicity.^5–7^

Among these candidates, bismuth- and antimony-based halides have recently reemerged as particularly compelling. Although their chloride and bromide derivatives were investigated decades ago for ferroic behavior,^8–11^ iodide analogues have only recently been recognized for their narrower band gaps and tunable electronic structures, features critical for next-generation optoelectronic devices.^12–14^ However, a persistent challenge lies in their strong tendency to form low-dimensional frameworks,^7,14–18^ where reduced lattice connectivity inhibits efficient charge transport. The limited availability of suitable organic cations capable of directing the assembly of higher-dimensional networks further complicates rational materials design. Even the use of longer-chain amines, which can stabilize 2D architectures,^13,14,19,20^ often results instead in the formation of lower-dimensional phases.

Despite these obstacles, low-dimensional halides remain highly relevant. Zero- and one-dimensional iodides exhibit rich and distinctive physical properties, enabling applications such as X-ray detection and scintillation.^21–23^ Moreover, many iodide-based hybrids display pronounced thermochromism–strong, reversible color changes induced by temperature variation–offering opportunities for thermal sensing, smart window technologies, and energy storage.^24–30^ Yet, despite decades of sporadic reports, the fundamental mechanism governing thermochromism in hybrid halides remains poorly understood, in part due to limited availability of structural data across temperature regimes.

Hybrid iodides represent the most prominent class of thermochromic halides.^16,25–27,31,32^ Bromide analogues, while known, typically exhibit much weaker thermochromism because their more rigid inorganic sublattices and shorter Bi/Sb–Br bonds limit lattice flexibility and shift absorption toward the ultraviolet region.^25^ By contrast, the inherently flexible Bi/Sb–I frameworks readily undergo octahedral distortions, halide reorganization, and I···I interactions, providing favorable platform for temperature-induced structural transformations that manifest as significant color changes.^25^ Not all thermochromic responses arise from phase transitions; usually subtle structural distortions or electronic reconfigurations within either the inorganic lattice or the organic chromophore modulate absorption and trigger color changes.^28,29,33^ Elucidating how these coupled inorganic–organic degrees of freedom shape thermochromism is therefore critical for establishing design principles for next-generation temperature-responsive and light-harvesting materials.

In this work, we demonstrate that a butylammonium-based organic–inorganic bismuth iodide, **(But)**_**3**_ **[BiI**_**6**_ **]**, exhibits complex thermochromic behavior and acts as a multiresponsive material to temperature stimuli, with its optical and dielectric properties governed by structural phase transitions. Furthermore, the discrete BiI_6_ units forming the inorganic framework display one-dimensional electronic characteristics, arising from their specific arrangement and I···I interactions between neighboring octahedra. The long-chain But^+^ cations act as molecular clips bridging the adjacent octahedra via N–H···I hydrogen bonds while simultaneously providing structural flexibility by adapting to changes in the BiI_6_ arrangement. Structural rearrangements associated with two phase transitions give rise to three polymorphic forms with distinct optical, dielectric, and electronic properties. Notably, the temperature-dependent color change (orange → green → orange upon cooling) is well reproduced by DFT calculations. The **I** → **II** phase transition drives dielectric switching, whereas the **II** → **III** transition induces second-harmonic generation (SHG) response. All these effects, highlighting the pronounced thermal responsiveness of **(But)**_**3**_ **[BiI**_**6**_ **]**, were elucidated using X-ray diffraction, spectroscopic techniques (linear, nonlinear, and dielectric), and DFT calculations.

## Experimental Methods

### Synthesis

**(But)**_**3**_ **[BiI**_**6**_ **]** was obtained by mixing stoichiometric amounts of 4 mmol butylamine (≥99%) and 1 mmol Bi_2_O_3_ (≥99.90%, Acros Organics) in 7 mL of concentrated hydroiodic acid (54%, Acros Organic). The solution was heated to 70 °C with constant stirring until a clear red solution was obtained. After slow evaporation of the solution (cooling rate 2 °C/h), orange crystalline plates appeared. To confirm the elemental composition and chemical states of the material, SEM-EDS analysis was employed (Figure S17).

### Thermal Analysis

DSC was measured using Metler Toledo DSC3 instrument. Powder sample was cooled and heated in the range of 120–450 K, with 10 K/min ramp rate. DSC and TGA curves are illustrated on Figures 1a, and S2–S4 respectively.

**Figure 1:**
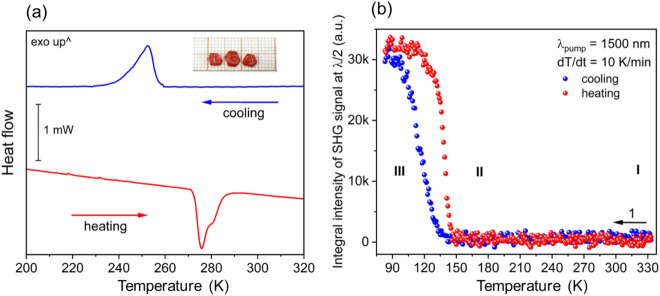
Results of (a) DSC and (b) SHG in **(But)_3_[BiI_6_]**. DSC curves and integral intensities of the SHG signal are plotted as a function of temperature for cooling and heating runs.

### Single-Crystal X-ray Diffraction

Single-crystal X-ray diffraction (SCXRD) data were obtained using an Xcalibur four-circle diffractometer (Oxford Diffraction) equipped with an Atlas CCD detector and a Mo Kα radiation source (λ = 0.7107 Å). Absorption effects were corrected using a multiscan method implemented in *CrysAlis* PRO 1.171.42.49 (Rigaku Oxford Diffraction, 2022). Empirical absorption correction using spherical harmonics was implemented in SCALE3 ABSPACK scaling algorithm. The crystal structures were solved with SHELXT within the Olex2 1.5 ^34,35^ software package and subsequently refined using SHELXL.^36^ Hydrogen atom parameters were treated using constrained refinement. Single crystal data were collected in wide range of temperatures (100–300 K) with measurement every 10 K to record lattice parameter changes. Solution and refinement of **(But)**_**3**_ **[BiI**_**6**_ **]** were conducted at 295 K (phase **I**, *Pnnm*, *a* = 14.331(4) Å, *b =* 24.077(6) Å, 8.802(3) Å, *Z =* 4, *V =* 3037.2(2) Å^3^, *R*_1_ = 0.049, *wR*_2_ = 0.189), 200 K (phase **II**, *Pccn*, *a =* 13.663(4) Å, *b =* 24.402(6) Å, *c =* 17.517(5) Å, *Z =* 8, *V =* 5840(3) Å^3^, *R*_1_ = 0.044, *wR*_2_ = 0.097), and 120 K (phase **III**, *P*2_1_2_1_2, *a =* 13.593(4) Å, *b =* 24.052(6) Å, *c =* 17.528(5) Å, *Z =* 8, *V =* 5731(3) Å^3^, *R*_1_ = 0.027, *wR*_2_ = 0.045), respectively. Nonstandard settings of space groups were chosen to allow straightforward comparison of unit cell parameters and structural evolution across all three phases. Experimental details, selected geometric parameters and hydrogen bonds are listed in Tables S1–S6 in the SI.

Hirshfeld surface analysis and two-dimensional fingerprint plots were performed to investigate intermolecular interactions in two low-temperature phases of the title compound (200 and 120 K) using CrystalExplorer (ver. 17.5).^37^

### Optical Observations of Thermochromic Behavior

Temperature-dependent changes in thermochromic **(But)**_**3**_ **[BiI**_**6**_ **]** crystals were studied using a polarizing microscope Olympus BX53 equipped with a LINKAM THM–600 cooling/heating stage. The temperature scanning (heating/cooling) rate was set to 15 K/min, and it was reduced to 1 K/min in the area of the phase transitions. The reversibility and stability of thermochromic behavior were confirmed over five consecutive heating/cooling cycles performed on the same crystal, on a one-year-old powder sample, and on a defected crystal, as illustrated in Figure S18 (1–5, (a) and (b), respectively) in the Supporting Information.

### Powder X-ray Diffraction

At room temperature, powder X-ray diffraction (PXRD) of the ground samples was measured using an PANalytical X’pert Pro diffractometer (in Bragg–Brentano geometry) with a PIXcel line detector and Cu *K*α radiation (λ = 1.5418 Å).

The diffractograms confirmed the purity of powders used in dielectric, thermal, and absorption measurements (Figures S11, S12). Additionally, an Oxford Cryosystems Phenix cryostat was used to collect diffractograms at different temperatures: 295, 200, and 120 K.

### Dielectric Spectroscopy

The complex dielectric response measurements were conducted from 100 to 280 K using an Agilent E4980A Precision LCR Meter in the 135 Hz – 2 MHz frequency range. Due to stress-induced cracking of a single crystal during phase transition, measurements were conducted on pressed pellets coated with a gold electrode using a Quorum Q150T sputter coating system (geometrical parameters: S = 40 mm^2^, d = 0.9–1 mm).

### Absorption Measurements

Absorption spectra were recorded using an Agilent Cary 5000 spectrophotometer in the wavelength range of 240–800 nm, with a step size of 0.25 nm.

### Second Harmonic Generation (SHG) Measurements

To verify the symmetry of the temperature-dependent crystal phases of **(But)**_**3**_ **[BiI**_**6**_ **]** and to characterize its phase-transition behavior, temperature-resolved second-harmonic generation (TR-SHG) measurements were conducted. The powdered sample was irradiated with a 1500 nm femtosecond laser while the temperature was cycled between 333 and 83 K at a rate of 10 K/min.

### Computation Methods

First-principles calculations based on density functional theory (DFT) were carried out using the QUANTUM ESPRESSO software package (version 6.8).^37–39^ The exchange-correlation energy was treated within the generalized gradient approximation (GGA)^40^ with the Perdew–Burke–Ernzerhof (PBE)^41^ functional. Electronic properties were calculated using scalar and fully relativistic projector augmented-wave (PAW) pseudopotentials generated by A. Dal Corso.^42^ The kinetic energy cutoff for charge density was set to 400 Ry, and the plane-wave cutoff for the wave function was 40 Ry. The Brillouin zone was sampled using a 5 × 5 × 5 Γ-centered Monkhorst–Pack grid for all calculations.^43^ To improve the accuracy of the electronic structure, the self-consistent field (SCF) convergence threshold was tightened to 10^–8^ Ry. For nonself-consistent (NSCF) calculations, the tetrahedron method was applied for Brillouin zone integration.^44^ The Brillouin zone path was generated using the SeeK–path tool^45^ and defined as Γ—X—S—Y—Γ—Z—U—R—T—Z|X—U|Y—T|S—R|, where Γ (0.0 0.0 0.0), X (0.5 0.0 0.0), S (0.5 0.5 0.0), Y(0.0 0.5 0.0), Z (0.0 0.0 0.5), U (0.5 0.0 0.5), R (0.5 0.5 0.5), and T (0.0 0.5 0.5) correspond to the conventional high-symmetry points. The effective masses of charge carriers were evaluated using the parabolic approximation of the band dispersion, based on the second derivative of the energy E­(*k*) with respect to the crystal momentum *k*, according to the following equations:

1 $$\frac{1}{m_{h}^{*}}=\frac{1}{\hbar^{2}}\frac{d^{2}}{dk^{2}}E_{VB\;max}\left(k\right),\frac{1}{m_{e}^{*}}=\frac{1}{\hbar^{2}}\frac{d^{2}}{dk^{2}}E_{CB\;min}\left(k\right)$$

The electronic band gaps were calculated from GGA/PBE+SOC. The GGA/PBE functional provides surprisingly good agreement with experimentally determined optical band gaps in 0D hybrids built of isolated face-sharing double octahedra like Cs_3_Bi_2_I_9_ and MA_3_Bi_2_I_9_.^46,47^ In hybrids containing single BiI_6_ octahedra, the inclusion of spin–orbit coupling (SOC) is essential to correlate the results of calculations with those obtained experimentally. On the other hand, pure PBE or HSE06 hybrid functionals tend to overestimate the band gap. For example, in Cs_2_NaBiI_6_, GGA/PBE predicts a band gap of 2.23 eV, HSE06+SOC yields 2.43 eV,^47^ while GGA/PBE+SOC reduces it to 1.68 eV^48^—very close to the experimental value of 1.66 eV.

### Phase Transitions in **(But)_3_[BiI_6_]**

**(But)**_**3**_ **[BiI**_**6**_ **]** crystallizes as dark-orange plates that remain stable under ambient conditions after drying from the crystallization solution. TGA analysis (Figure S4) confirms the stability of the crystal up to 500 K, with the onset of decomposition at approximately 525 K. Upon cooling, two low-temperature (LT) polymorphs become stabilized. Differential scanning calorimetry (DSC) measurements reveal a single-phase transition (PT) at 260 K during cooling and at 275 K upon heating. Second-harmonic generation (SHG) measurements indicate an additional transition from a centrosymmetric to a noncentrosymmetric space group at approximately 130 K during the cooling cycle, accompanied by a large thermal hysteresis of 22 K. The absence of this second transition in the DSC trace is attributed to the limited sensitivity of the instrument below 150 K. Figure 1a shows the thermal anomalies associated with the **I** ↔ **II** PT. The temperature dependence of the integral SHG intensity (λ_max_ = 750 nm), confirming the **II** ↔ **III** PT, is plotted in Figure 1b (for experimental SHG spectra see also Figures S14 and S15 in SI).

During the initial cooling run from 333 K, no SHG signal was detected for crystal phases **I** and **II**, which confirms their centrosymmetric nature. Upon further cooling, a sharp onset of SHG activity is observed below 130 K, indicating a structural phase transition to the noncentrosymmetric phase **III**. In the subsequent heating cycle, the SHG signal persists up to approximately 152 K before vanishing completely, indicating the return to the centrosymmetric phase **II**. This sharp discontinuity in the nonlinear optical response, coupled with the observation of a broad thermal hysteresis spanning approximately 22 K between cooling and heating cycles, evidence the first order (discontinuous) character of this phase transition. To quantify the second-harmonic conversion efficiency of phase **III**, a Kurtz–Perry (Graja) powder test^49,50^ was performed comparing the SHG signal of **(But)**_**3**_ **[BiI**_**6**_ **]** at 83 K with that of potassium dihydrogen phosphate (KDP) at 293 K (see Figure S16 in SI), using identical beam parameters and experimental geometry to enable direct comparison. The relative SHG efficiency of **(But)**_**3**_ **[BiI**_**6**_ **]** in phase **III** was found to be modest, approximately 0.036 versus that of KDP.

### Crystal Structure

The SC-XRD analysis revealed that the **(But)**_**3**_ **[BiI**_**6**_ **]** hybrid is built of isolated BiI_6_ octahedra separated by protonated butylammonium counterions. The successive phase transitions stabilize three orthorhombic polymorphs: two centrosymmetric phases, **I** of *Pnnm* and **II** of *Pccn* space groups, and noncentrosymmetric phase **III** of *P*2_1_2_1_2 symmetry. The symmetry of the subsequent phases was also confirmed by low-temperature powder X-ray diffraction, as shown in Figure S13.

The structure of phase **I** is characterized by a large degree of disorder. At room temperature, all atomic positions of three independent butylammonium cations are split into at least two symmetry-equivalent sites with identical occupancy. Two But^+^ adopt an all-*anti* conformation, whereas one is a *gauche* (*anti*-*anti*-*gauche*) conformer. Large displacement parameters within the inorganic part confirm temperature-induced disorder of the whole structure. The basic building units of **(But)**_**3**_ **[BiI**_**6**_ **]** at RT are shown in Figure S1.

Cation ordering at 260 K induces a phase transition that markedly alters the crystal packing. The most pronounced changes are observed within (*a, b*) planes. Along the *a-*axis, the unit cell shortens from 14.29 to 13.7 Å and along the *b-*axis it increases abruptly from 24.0 to 24.7 Å upon cooling, at the transition point. Concurrently, the *c* parameter remains nearly unchanged throughout both phases. The discontinuous change in unit-cell volume confirms first-order character of the **I–II** PT. The rearrangement of the crystal structure along with the relative changes of lattice parameters is shown in Figure 2 whereas temperature evolution of lattice parameters is shown in Figure S5. Cation ordering is triggered by the formation of N–H···I hydrogen bonds at the organic–inorganic interface, with donor-to-acceptor distances ranging from 3.63 to 3.89 Å at 200 K (Table S5), effectively hindering rotational motion of the carbon chains. The release of the additional volume in the structure leads to significant shifts of BiI_6_ octahedra within (*a, b*) planes and changes in interoctahedral I···I interactions.

**Figure 2:**
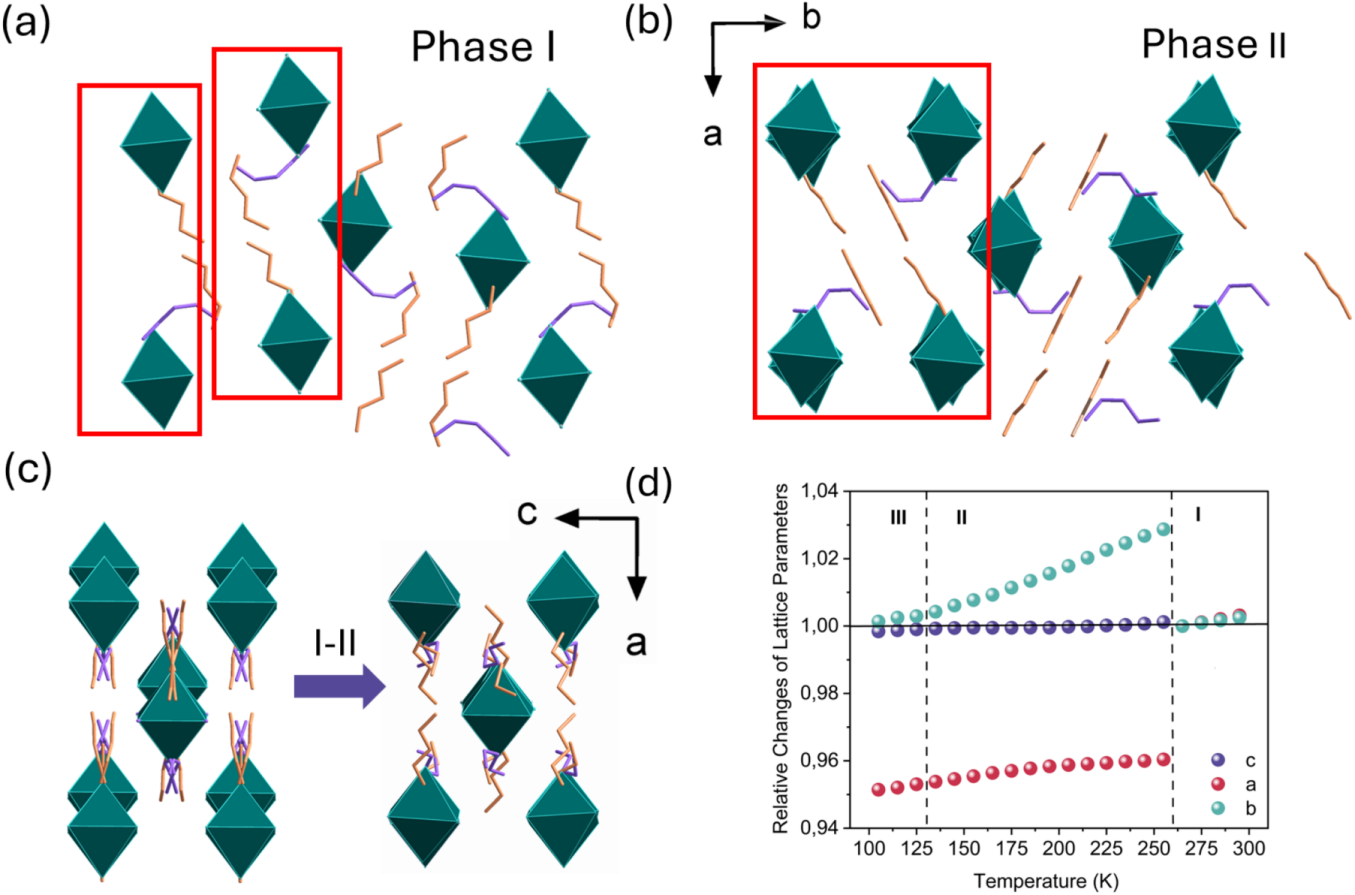
Crystal structure transformation across **I–II** PT. Unit cell packing along the *c*-axis for high-temperature phase at 295 K and low-temperature phase at 200 K. The view along the *c*-direction in (a) phase **I**, *T* = 295 K, (b) phase **II**, *T* = 200 K; (c) packing along *b*-direction, (d) relative changes of lattice parameters with temperature lowering.

The shortest van der Waals I···I contacts are formed along the *b*-axis (I4–I4 = 4.146(1) Å at 200 K) between symmetry-related units and along the *c*-axis with I4···I6 separation of 4.371(12) Å at 200 K. These contacts define a pseudoribbon motif composed of BiI_6_ octahedra extending along *c* (Figures 3a and 7). The exceptionally short distance between iodides is maintained by the butylammonium cations, which act as a molecular “clip” linking the octahedra through N–H···I hydrogen bonds (Figure 3b) with comparable N···I distances of ∼3.60–3.63 Å, and contributing significantly to the stability of this structural motif. Notably, the octahedral distortion parameters remain essentially unchanged (Table S7). Both the bond-length distortion and angle variance exhibit only minor variations, from 0.014 to 0.012 and from 3.00 to 4.14 °^2^, respectively. These observations indicate that the crystal structure of phase **II** is governed primarily by packing optimization, rather than by local distortions of the coordination polyhedra.

**Figure 3:**
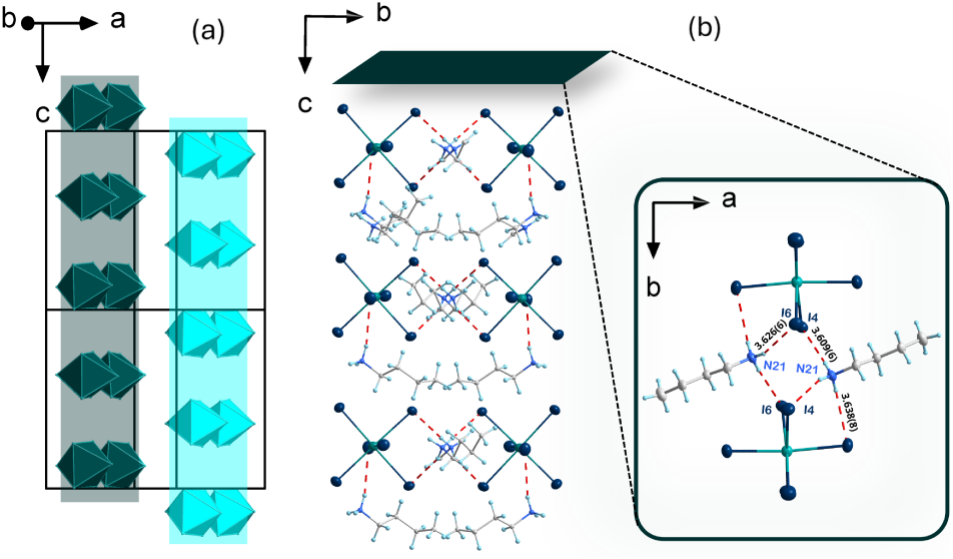
Crystal structure in phase **II**, *T* = 200 K. Pseudodouble-chains of BiI_6_ octahedra interacting through van der Waals I···I contacts along *c*-axis (a), hydrogen bonds fastening octahedra in a ribbon (b).

The low-temperature asymmetric unit at 200 K (Figure S1) comprises a single isolated octahedron with all iodide atoms symmetry-independent, together with three positionally ordered butylammonium cations. However, at this temperature the carbon atoms of But^+^ exhibit relatively large displacement parameters, indicating that complete ordering has not yet been achieved in phase **II**.

The second phase transition at 130 K is related to a *Pccn* → *P*2_1_2_1_2 symmetry reduction. Although the group-subgroup relationship is maintained and only a slight inflection in the temperature dependence of the lattice parameters appears near the transition, which might suggest continuous transformation, the broad temperature hysteresis of SHG signal implies a first-order PT. In contrast to the PT at 260 K, the low-temperature transformation proceeds without significant rearrangement of either the inorganic framework or the organic conformations. Instead, the asymmetric unit doubles and contains two octahedra and six cations (four all-*anti*, two *gauche*).

A structural signature of this transition at the molecular level is the change in the position of the *gauche* cation (N31) at 200 K. The atomic displacement amplitude |u| calculated in Amplimodes^51,52^ reaches approximately 0.24 Å for each atom-about twice that of the remaining cations in the structure. The torsion angles of N31 But^+^ decrease upon cooling, from −175.6° (N31–C32–C33–C34) and 78.6° (C35–C34–C33–C32) at 200 K to −167.7° and 59°, respectively (Table S8). While the former remains close to the *anti*-conformation, the latter shifts toward a more compact *gauche* arrangement. These subtle changes in molecular conformation, shown in Figure S9, serve as the driving, displacive force for the LT phase transition, which effectively removes the inversion center. Although the dominant H···I and H···H interactions are preserved, the loss of inversion symmetry results in differentiation of previously equivalent environments, as reflected in the Hirshfeld fingerprint plots (Figures S8 and S9). Quantitative analysis of the Hirshfeld surfaces (Figure S10) confirms that the relative proportions of H···H, H···I, and I···I contacts remain nearly unchanged across the phase transition. Therefore, the structural transformation mainly affects the symmetry equivalence of the molecular environments rather than the overall balance of intermolecular interactions.

### Dielectric Response

Dielectric measurements were carried out during cooling and heating cycles at temperatures between 100 and 300 K and frequencies between 2 kHz and 2 MHz, in order to investigate possible relaxation processes and evaluate the impact of structural phase transitions on the dielectric properties of **(But)_3_[BiI_6_]**. Figure 4 shows the temperature dependence of the real part of the complex dielectric permittivity for the polycrystalline sample in the vicinity the low-temperature phase transitions during the cooling cycle. Details of the sample preparation can be found in the “Experimental” section. Two subtle anomalies can be identified in the ε′(T) dependence. The phase transition from phase **I** to phase **II** is associated with a step-like anomaly, with a dielectric increment (Δε′) of approximately 0.9 at 2 MHz. In contrast, the phase transition from phase II to phase III is only reflected by a slight change in the slope of the ε′(T) curve. No dielectric dispersion was observed over the investigated temperature and frequency ranges. The inset in Figure 4 shows an example of reversible dielectric switching between “ON” and “OFF” states at 2 MHz, and illustrates the results obtained from several consecutive measurement cycles performed on a polycrystalline sample. In the case of **(But)_3_[BiI_6_]**, no weakening of the dielectric signal was observed during cyclic processes, which proves the samples’ high thermal and electrical stability.

**Figure 4:**
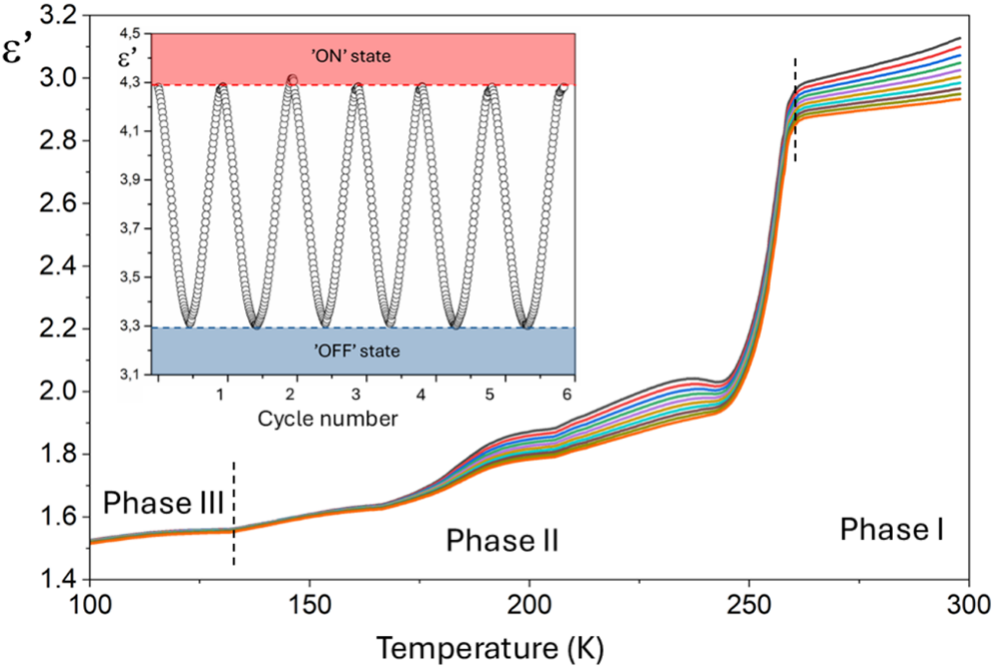
Temperature dependence of ε’ measured between 2.2 kHz and 2 MHz during a cooling cycle (⌀ 5 mm, d = 0.92 mm). The inset shows the dielectric signature of the phase transition between **I** and **II** variation of ε’ measured at 2 MHz as a function of temperature during cooling–heating cycles (⌀ 5 mm, d = 0.82 mm).

### Thermochromic Behavior

The thermochromic behavior of the single crystal was investigated using polarized optical microscopy under controlled cooling conditions. With the aid of a cooling stage, a distinct two-step color evolution was observed (see Supporting Information, Video S1). Upon initial cooling, the crystal exhibited a gradual transition from dark orange to a lighter orange shade, indicative of a continuous thermochromic response. Such behavior is commonly observed in iodide-based systems and can be attributed to the relatively soft and deformable Bi–I bonds, which enable subtle lattice contractions and modulate the band gap. However, at the temperature corresponding to the first phase transition (T_1_), a sharp and discontinuous color change from light orange to green occurs, reflecting an abrupt structural rearrangement. The green coloration persists throughout phase **II** and reverts to orange at ∼130 K, corresponding to the **II→III** phase transition.

To corroborate these observations, temperature-dependent diffuse reflectance measurements were performed on powdered samples over the range 140–303 K. Measurements at lower temperatures were limited by the experimental setup and could not be extended into phase **III**. A pronounced blue shift of the absorption edge (∼40 nm) was observed upon cooling, comparable to values previously reported for hybrid thermochromic materials.^29^ The observed color evolution is consistent with the temperature-dependent shift of the band gap. At *E*_g_ = 2.28 eV (≈544 nm), the crystal appears green, indicating that absorption predominantly removes higher-energy (blue) components while preserving transmission in the green region. Upon band gap reduction to *E*_g_ = 2.19 eV (≈566 nm), the absorption extends further into the green region, resulting in a shift of the transmitted light toward longer wavelengths and an orange appearance.

The sequence of color changes (orange → green → orange) is directly correlated with the structural phase transitions and associated lattice rearrangements. In particular, the anomaly in the *b* lattice parameter at T_1_, followed by its decrease upon further cooling and recovery to values comparable to those of phase **I** near 130 K, reflects changes in interoctahedral distances and I···I contacts. The unusual evolution of the band gap in phase **III** is further supported by density functional theory (DFT) calculations. Figure 5a–e and S18 show the thermal evolution of the absorption edge in phases **I** and **II**, temperature-induced changes in the *b*-lattice parameter related to the color changes, and color changes across the three polymorphs.

**Figure 5:**
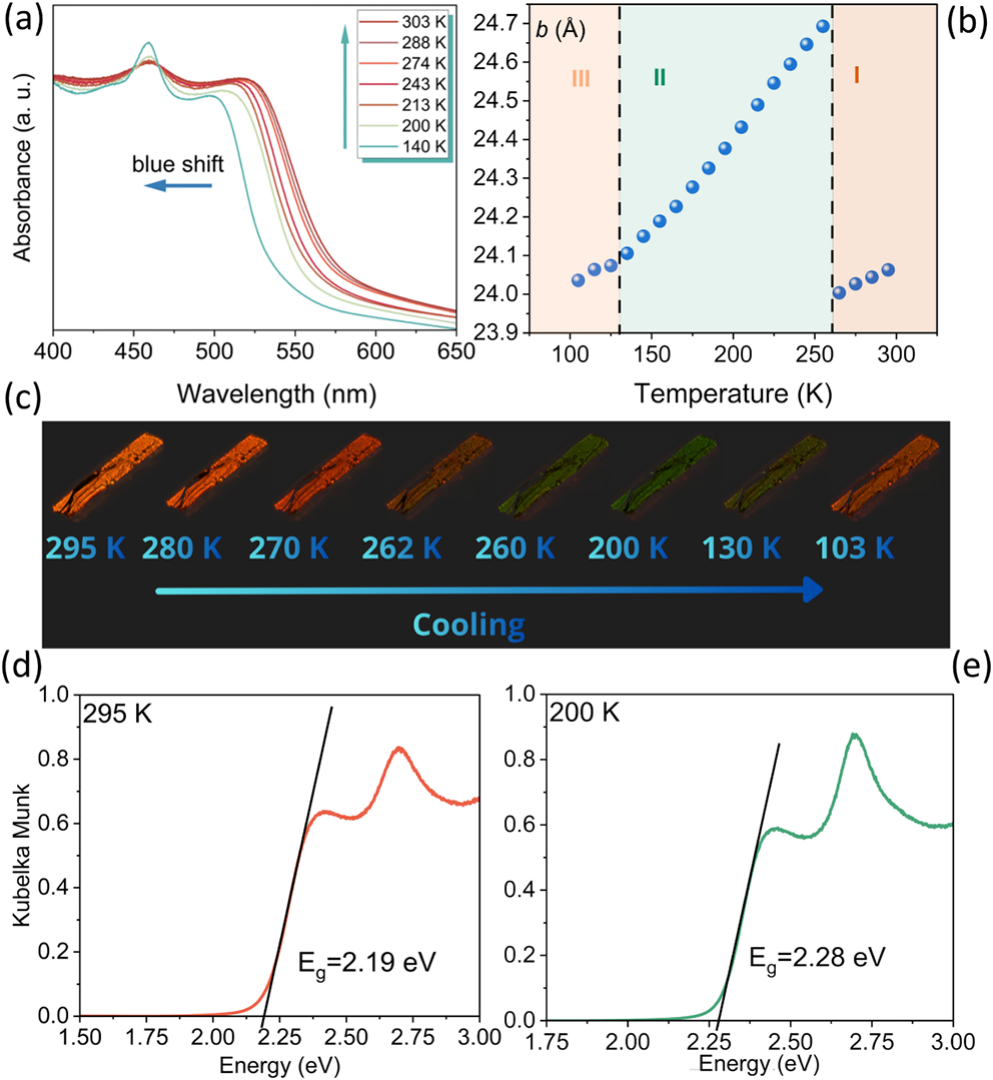
Absorption spectra across phase **I** and **II** for **(But)_3_[BiI_6_]** with blue shift of the absorption edge upon cooling (a); temperature evolution of the *b*-lattice parameter (b); thermochromic behavior of **(But)_3_[BiI_6_]** upon cooling across phases **I**, **II**, and **III** (c); band gap of **(But)_3_[BiI_6_]** calculated using the Kubelka–Munk function at 295 and 200 K (d, e).

### The Electronic Properties of **(But)_3_[BiI_6_]**

The band structure, partial density of states (PDOS), and carrier effective mass were calculated to determine the interplay between the butylammonium-based organic and inorganic bismuth iodide subsystems, in both ordered LT phases of **(But)**_**3**_ **[BiI**_**6**_ **]**.

Both GGA/PBE and GGA/PBE+SOC calculations reproduce the experimentally observed trends reasonably well, but the inclusion of spin–orbit coupling (SOC) proved essential for obtaining realistic band gap values. The calculated band structure of **(But)**_**3**_ **[BiI**_**6**_ **]** is presented in Figure 6.

**Figure 6:**
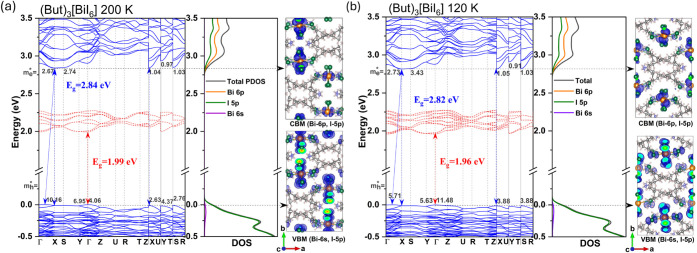
Scalar relativistic (blue solid line) and fully relativistic (red dashed line) band structures. (a) **(But)_3_[BiI_6_]**, 200 K; (b) **(But)_3_[BiI_6_]**, 120 K, with a scalar relativistic partial density of states (PDOS).

Based on calculated density of states (TDOS) and PDOS, the valence band maximum (VBM) originates predominantly from σ-antibonding interactions between Bi 6s and I 5p orbitals, while the conduction band minimum (CBM) is mainly composed of hybridized Bi 6p and I 5p states, which can be assigned as a p → p transition with (s-p hybridization) within the anionic BiI_6_ octahedra subsystem. The contribution of organic cations to the frontier orbitals is extremely small, but their influence is more indirect, mainly by affecting the distortion and spatial arrangement of the BiI_6_ units. Both LT phases display similar electronic behavior and are characterized by a direct band gap located at the Γ point. The VBM originates predominantly from σ-antibonding interactions between Bi 6s and I 5p orbitals, while the conduction band minimum (CBM) is mainly composed of hybridized Bi 6p and I 5p states. Calculations based on the structural model of phase **III**, performed using the GGA/PBE+SOC approach, predict a band gap of 1.96 eV at 120 K. In contrast, calculations for phase **II** yield a slightly higher value of 1.99 eV at 200 K, indicating an unusual narrowing of the band gap upon cooling. While such small differences are often within the intrinsic accuracy limits of the method, in this case they consistently reproduce the experimentally observed color change from green to orange across the **II** → **III** phase transition and confirm the blue shift of the absorption edge in phase **III**.

Two principal conclusions can be drawn from the combined DFT analysis and crystallographic data. First, the alignment of BiI_6_ octahedral pairs into columnar stacking motifs propagating along the *c*-axis gives rise to a quantum-wire-like electronic structure. This quasi-one-dimensional arrangement is stabilized by weak I···I van der Waals interactions and is consistent with the calculated band dispersion, charge density distribution, and effective mass anisotropy. The electronic charge density associated with the conduction band minimum (CBM, shown in blue) for **(But)**_**3**_ **[BiI**_**6**_ **]** at 200 K is presented in Figure 7. The computed effective masses confirm the anisotropic character of charge transport. The presence of the quantum-wire-like motif along the *c*-direction is manifested by the lowest effective masses along the Γ–Z and related *k*–paths (X–U, Y–T, S–R). Moreover, the calculations indicate that electrons possess significantly higher mobility than holes.

**Figure 7:**
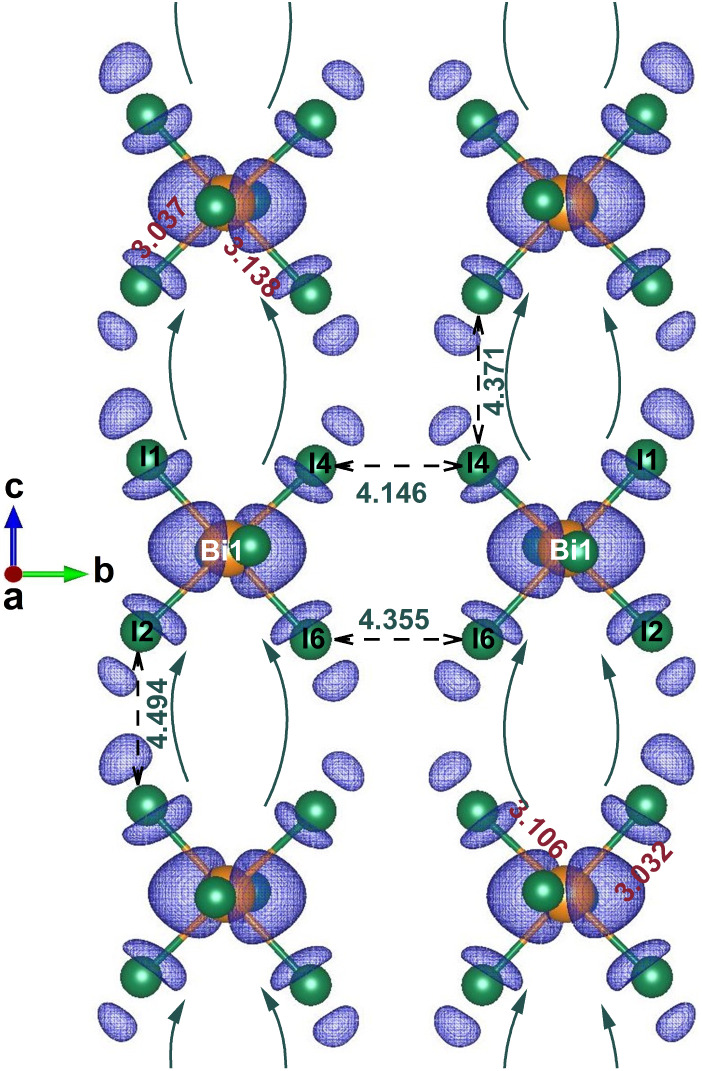
Quantum-wire-like motif along the *c*-direction in **(But)_3_[BiI_6_]** 200 K, with calculated electronic charge densities CBM (blue) and selected geometric parameters (Å).

Structurally, the shortest interoctahedral I···I contact occurs along the *b*-direction, with I4···I4 distances of 4.146(2) Å at 200 K and 4.095(6) Å at 120 K. Nevertheless, charge transport along this direction is less favorable because it requires transitions between adjacent ribbons separated by bulky organic cations. Consequently, the organic components act as wide-band gap barriers that confine charge carriers within the inorganic chains and restrict their motion perpendicular to the stacking direction.

The second notable electronic feature is the unusual temperature dependence of the band gap, which narrows upon cooling. Experimentally, this behavior is manifested as a color change from green to orange during the transition from phase **II** to phase **III**. This effect can be attributed to the shortening of interoctahedral distances between BiI_6_ units along the *b*-axis, approaching values characteristic of the high-temperature orange phase (**I**).

The transition from phase **I** to phase **II** involves a pronounced lattice expansion along the *b* direction, with Δ ≈ 0.7 Å at the phase boundary. Upon further cooling, a positive thermal expansion within phase **II** restores the interoctahedral separation to that typical of phase **III** near the second phase transition at approximately 130 K, leading to the reappearance of the orange color.

## Conclusions

We report a simple organic–inorganic hybrid of composition **(But)**_**3**_ **[BiI**_**6**_ **]** that exhibits complex temperature-responsive optical and dielectric properties. Two reversible phase transitions, occurring at 260/274 K and 130/150 K upon cooling/heating, give rise to three orthorhombic polymorphs: phase **I** (*Pnnm*), phase **II** (*Pccn*), and the noncentrosymmetric phase **III** (*P*2_1_2_1_2).

A molecular-level mechanism of these structural phase transitions is proposed (Scheme 1), highlighting their direct impact on the dielectric response, optical properties, and the observed dual thermochromic behavior. The combination of discrete BiI_6_ anionic units with highly anisotropic, long-chain butylammonium cations results in a temperature-sensitive and structurally flexible framework, governed by N–H···I hydrogen bonding and van der Waals I···I interactions. The unusual reversible color changes (orange → green → orange) across phases **I→III** are well reproduced by density-of-states calculations, demonstrating that DFT methods can reliably predict thermochromic behavior in molecular bismuth­(III) iodides. The coexistence of thermochromism, dielectric switching, and noncentrosymmetric functionality (SHG activity in phase **III**) establishes **(But)**_**3**_ **[BiI**_**6**_ **]** as a prototype compound demonstrating the feasibility of designing simple yet robust hybrid systems with intrinsically coupled optical, electrical, and nonlinear optical responses. Such a property combination is particularly promising for multifunctional, stimuli-responsive technologies, including temperature and optical sensing, switchable dielectrics, and frequency-doubling materials, within the broader context of lead-free and environmentally sustainable optoelectronic and photonic applications.

**Scheme 1:**
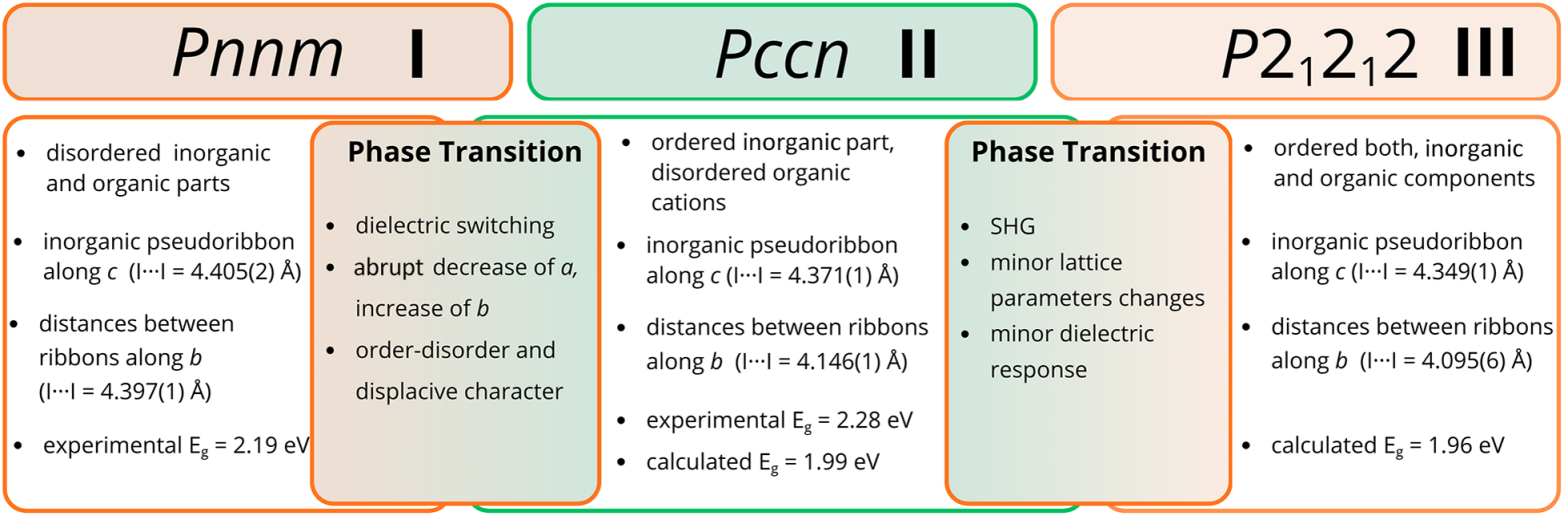
A Scheme Summarizing the Phase Transition-Induced Structural Changes with Connections to Optical/Dielectric/SHG Behaviors

## Supplementary Material





## Notes

Crystallographic data and computation methods (Tables S1–S9); powder X-ray diffraction, basic building blocks, hydrogen bonds, Hirshfeld surfaces and fingerprints, DSC, TGA, lattice parameters, I···I interactions, SHG spectra, mechanistic schemes, thermochromic behavior, SEM-EDS results (Figures S1–S18; Scheme S1); CIF files for structures at 295, 200, and 120 K; and (PDF)
color changes across the phase transitions recorded on a single crystal (AVI)
