# Supplementary material for: A Multi-Responsive Bismuth-Based Hybrid Exhibiting Dual Thermochromism, SHG Activity, and Dielectric Switching Induced by Sequential Phase Transitions
Source: Inorg Chem. 2026 Jul 16;65(30):17729–37. doi: 10.1021/acs.inorgchem.6c02522 (PMC13439665; doi:10.1021/acs.inorgchem.6c02522)
Supplement: Supplementary file 1 [file ic6c02522_si_001.pdf]

A Multi–Responsive Bismuth–Based Hybrid Exhibiting Dual Thermochromism, SHG Activity and Dielectric Switching Induced by Sequential Phase Transitions

Magdalena N. Rowińska[a], Oleksandr Korolevych[a], Tamara J. Bednarchuk[a], Jan K. Zaręba[b], Anna Piecha-Bisiorek[c], Anna Gągor[a]

[a] Institute of Low Temperature and Structure Research, Polish Academy of Sciences, Okólna 2, 50-422 Wrocław, Poland

[b] Institute of Advanced Materials, Wrocław University of Science and Technology, Wrocław, 50–370 Poland

[c] Faculty of Chemistry, University of Wrocław, F. Joliot-Curie 14, Wrocław 50-383, Poland

Table S1. Experimental details

	$(But)_3[BiI_6]$		
Chemical formula	$BiI_6 \cdot 3(C_4H_{12}N)$		
Crystal system, space group	Orthorhombic, *Pnnm*	Orthorhombic, *Pccn*	Orthorhombic, $P2_12_12$
**Temperature (K)**	**295**	**200**	**120**
a, *b*, *c* (Å)	14.331 (4), 24.077 (6), 8.802 (3)	13.663 (4), 24.402 (6), 17.517 (5)	13.593 (4), 24.052 (6), 17.528 (5)
V (Å^3)	3037.2 (15)	5840 (3)	5731 (3)
Z	4	8	8
Radiation type	Mo Kα		
μ (mm^{-1})	11.90		
Crystal size (mm)	0.25 × 0.1 × 0.07		
**Data collection**			
Diffractometer	Xcalibur, Atlas		
Absorption correction	Multi-scan *CrysAlis PRO* 1.171.42.49 (Rigaku Oxford Diffraction, 2022) Empirical absorption correction using spherical harmonics, implemented in SCALE3 ABSPACK scaling algorithm.		
No. of measured, independent and observed [I > 2σ(I)] reflections	44105, 4240, 1756	29989, 7185, 4820	74705, 11708, 8951
R_{int}	0.062	0.041	0.057
$(sin\theta/\lambda)_{max}$ (Å^{-1})	0.692	0.689	0.625
**Refinement**			
$R[F^2 > 2\sigma(F^2)]$, $wR(F^2)$, S	0.049, 0.189, 0.98	0.044, 0.097, 1.05	0.027, 0.045, 0.99
No. of reflections	4240	7185	11708
No. of parameters	46	205	266
No. of restraints	32		108

$\Delta\rho_{max}$, $\Delta\rho_{min}$ (e Å^{-3})	1.38, -0.73	1.60, -1.25	1.14, -0.89
Absolute structure/ parameter			Refined as an inversion twin, 0.49 (2)

Computer programs: *CrysAlis PRO* 1.171.42.49 (Rigaku OD, 2022), *SHELXL* 2018/3 (Sheldrick, 2015), Olex2 1.5 (Dolomanov *et al.*, 2009).

Table S2. Selected geometric parameters (Å, º) 295 K

Bi1—I1	3.0308 (12)	Bi1—I3	3.1341 (12)
Bi1—I1^{i}	3.0308 (12)	Bi1—I3^{i}	3.1342 (12)
Bi1—I2	3.064 (2)	Bi1—I4	3.1197 (18)

I1—Bi1—I1^{i}	91.99 (5)	I1—Bi1—I4	90.57 (4)
I1—Bi1—I2	92.69 (4)	I2—Bi1—I3	88.89 (4)
I1^{i}—Bi1—I2	92.69 (4)	I2—Bi1—I3^{i}	88.89 (4)
I1—Bi1—I3	177.82 (4)	I2—Bi1—I4	175.30 (5)
I1—Bi1—I3^{i}	89.43 (4)	I3—Bi1—I3^{i}	89.10 (4)
I1^{i}—Bi1—I3^{i}	177.82 (4)	I4—Bi1—I3^{i}	87.77 (3)
I1^{i}—Bi1—I3	89.43 (4)	I4—Bi1—I3	87.77 (3)
I1^{i}—Bi1—I4	90.57 (4)		

Symmetry code(s): (i) *x*, *y*, -*z*+1.

Table S3. Selected geometric parameters (Å, º) 200 K

Bi1—I1	3.0323 (8)	Bi1—I4	3.1061 (8)
Bi1—I2	3.0366 (8)	Bi1—I5	3.0544 (11)
Bi1—I3	3.1025 (11)	Bi1—I6	3.1377 (8)

I1—Bi1—I4	89.74 (3)	I2—Bi1—I5	90.435 (19)
I1—Bi1—I6	178.32 (2)	I3—Bi1—I4	89.477 (18)
I1—Bi1—I3	91.831 (19)	I3—Bi1—I6	86.521 (18)
I1—Bi1—I2	91.59 (3)	I4—Bi1—I6	89.90 (3)
I1—Bi1—I5	91.999 (19)	I5—Bi1—I4	86.691 (18)
I2—Bi1—I4	176.876 (19)	I5—Bi1—I6	89.622 (18)
I2—Bi1—I6	88.86 (3)	I5—Bi1—I3	174.57 (2)
I2—Bi1—I3	93.308 (19)		

Table S4. Selected geometric parameters (Å, º) 120 K

Bi1—I1	3.035 (3)	Bi2—I7	3.036 (3)
Bi1—I2	3.026 (3)	Bi2—I8	3.032 (3)
Bi1—I3	3.097 (4)	Bi2—I9	3.099 (4)
Bi1—I4	3.103 (3)	Bi2—I10	3.101 (3)
Bi1—I5	3.055 (3)	Bi2—I11	3.056 (3)
Bi1—I6	3.145 (3)	Bi2—I12	3.138 (3)
		I11—I8^{i}	4.093 (4)

I1—Bi1—I6	177.72 (10)	I7—Bi2—I11	93.18 (10)
I1—Bi1—I3	90.67 (9)	I7—Bi2—I12	177.99 (10)
I1—Bi1—I4	89.11 (9)	I7—Bi2—I9	91.06 (10)
I1—Bi1—I5	93.35 (9)	I8—Bi2—I10	176.53 (10)
I2—Bi1—I6	89.06 (9)	I8—Bi2—I7	91.47 (9)
I2—Bi1—I3	94.67 (10)	I8—Bi2—I11	90.15 (9)
I2—Bi1—I4	176.28 (10)	I8—Bi2—I12	89.19 (9)
I2—Bi1—I1	91.49 (9)	I8—Bi2—I9	94.25 (9)
I2—Bi1—I5	89.72 (10)	I9—Bi2—I10	89.19 (9)
I3—Bi1—I6	87.07 (9)	I9—Bi2—I12	87.00 (9)
I3—Bi1—I4	89.00 (9)	I10—Bi2—I12	90.53 (8)
I4—Bi1—I6	90.48 (8)	I11—Bi2—I10	86.39 (9)
I5—Bi1—I6	88.87 (9)	I11—Bi2—I12	88.71 (9)
I5—Bi1—I3	173.97 (10)	I11—Bi2—I9	173.81 (10)
I5—Bi1—I4	86.57 (9)	Bi2—I11—I8^{i}	152.03 (11)
I7—Bi2—I10	88.93 (9)		

Symmetry code(s): (i) *x*-1/2, -*y*+1/2, -*z*+1.

Table S5. Selected hydrogen-bond parameters 200 K

D—H···*A*	*D*—H (Å)	H···*A* (Å)	*D*···*A* (Å)	*D*—H···*A* (°)
N11—H11A···I2^{ii}	0.91	3.03	3.648 (8)	126.9
N11—H11A···I5^{iii}	0.91	2.93	3.680 (8)	141.1
N11—H11B···I1	0.91	2.90	3.694 (8)	146.4
N11—H11C···I6^{ii}	0.91	2.98	3.886 (8)	172.4
N21—H21A···I6^{i}	0.91	2.76	3.626 (7)	159.0
N21—H21B···I3^{i}	0.91	2.88	3.638 (8)	141.6
N21—H21C···I4	0.91	2.70	3.609 (7)	175.7
N31—H31A···I5	0.91	2.82	3.717 (8)	168.1
N31—H31B···I3^{iv}	0.91	2.82	3.608 (9)	145.7
N31—H31C···I1^{v}	0.91	3.12	3.805 (9)	133.3

N31—H31C···I2	0.91	3.16	3.838 (10)	133.3

Symmetry code(s): (i) -*x*+1/2, -*y*+3/2, *z*; (ii) -*x*+1/2, *y*, *z*+1/2; (iii) -*x*+1, -*y*+1, -*z*+1; (iv) *x*+1/2, -*y*+1, -*z*+1/2; (v) -*x*+1/2, *y*, *z*-1/2.

Table S6. Selected hydrogen-bond parameters 120 K

D—H···*A*	*D*—H (Å)	H···*A* (Å)	*D*···*A* (Å)	*D*—H···*A* (°)
N11—H11A···I1	0.91	2.90	3.71 (2)	149.5
N11—H11B···I12	0.91	2.94	3.84 (2)	170.7
N11—H11C···I11^{ii}	0.91	2.76	3.59 (2)	152.6
N11—H11C···I8	0.91	3.08	3.606 (16)	118.3
N21—H21A···I6^{iii}	0.91	2.79	3.62 (2)	151.2
N21—H21B···I3^{iii}	0.91	2.72	3.56 (2)	154.9
N21—H21C···I4	0.91	2.75	3.63 (2)	163.0
N31—H31A···I5^{iv}	0.91	2.80	3.683 (15)	163.3
N31—H31B···I3	0.91	2.88	3.606 (16)	137.5
N31—H31C···I7^{v}	0.91	3.00	3.735 (15)	138.5
N31—H31C···I2^{iv}	0.91	3.18	3.795 (15)	127.2
N41—H41A···I2^{i}	0.91	2.96	3.699 (16)	139.3
N41—H41A···I5	0.91	3.11	3.69 (2)	123.1
N41—H41B···I6^{i}	0.91	3.01	3.82 (2)	149.0
N41—H41C···I7^{ii}	0.91	2.88	3.685 (19)	148.1
N51—H51A···I10	0.91	2.67	3.56 (2)	167.9
N51—H51B···I9^{iii}	0.91	2.98	3.64 (2)	130.4
N51—H51C···I12^{iii}	0.91	2.76	3.65 (2)	165.3
N61—H61A···I9^{v}	0.91	3.02	3.582 (16)	121.6
N61—H61A···I1	0.91	3.13	3.897 (15)	143.6
N61—H61B···I8^{v}	0.91	3.08	3.955 (15)	163.4
N61—H61C···I11	0.91	2.97	3.697 (15)	137.6

Symmetry code(s): (i) *x*+1/2, -*y*+1/2, -*z*+2; (ii) *x*+1/2, -*y*+1/2, -*z*+1; (iii) -*x*+1, -*y*, *z*; (iv) *x*-1/2, -*y*+1/2, -*z*+2; (v) *x*-1/2, -*y*+1/2, -*z*+1.

Table S7. Octahedral distortion

	**295**	**200**	**120**	
**Average bond length (Å)**	3.0856	3.0783	3.0771	3.0770
**Polyhedral volume (Å^3)**	39.1209	38.8222	38.7624	38.7624
**Bond angle variance (deg.^{2})**	3.0037	4.1705	5.2348	5.5033
**Distortion index (bond length)**	0.01415	0.01210	0.01149	0.01237

The irregularity of octahedra can be described by distortion index, D: $D = \frac{1}{6}\sum_{i=1}^{6}\frac{|l_i - l_{av}|}{l_{av}}$, where l_i is the distance from Co or Zn central atom to the ith coordinating atoms, and l_{av} – average Bi–X bond length within the octahedron.

The bond angle variance, σ²: $\sigma^2 = \frac{1}{m-1}\sum_{i=1}^{m}(\phi_i - \phi_0)^2$, where m is (number of faces in the octahedron, 8) × 3/2 (number of bond angles), ϕ_i- ith bond angles of the Bi octahedron, ϕ_0 - the ideal bond angle for a regular octahedron (90°).

Table S8. Torsion Angles(º) in 200 K

N11—C12—C13—C14	-179.7 (10)	C15—C14—C13—C12	174.1 (12)
N21—C22—C23—C24	-179.6 (8)	C22—C23—C24—C25	-176.3 (8)
N31—C32—C33—C34	-175.6 (12)	C35—C34—C33—C32	78.6 (18)

Table S9. Torsion Angles (º) in 120 K

N11—C12—C13—C14	176.3 (17)	N41—C42—C43—C44	174.0 (17)
C12—C13—C14—C15	161.0 (19)	C42—C43—C44—C45	171.4 (18)
N21—C22—C23—C24	-177.3 (15)	N51—C52—C53—C54	179.8 (15)
C22—C23—C24—C25	-179.3 (13)	C52—C53—C54—C55	171.6 (14)
N31—C32—C33—C34	-167.7 (15)	N61—C62—C63—C64	-173.6 (14)
C32—C33—C34—C35	59 (2)	C62—C63—C64—C65	-85.0 (17)

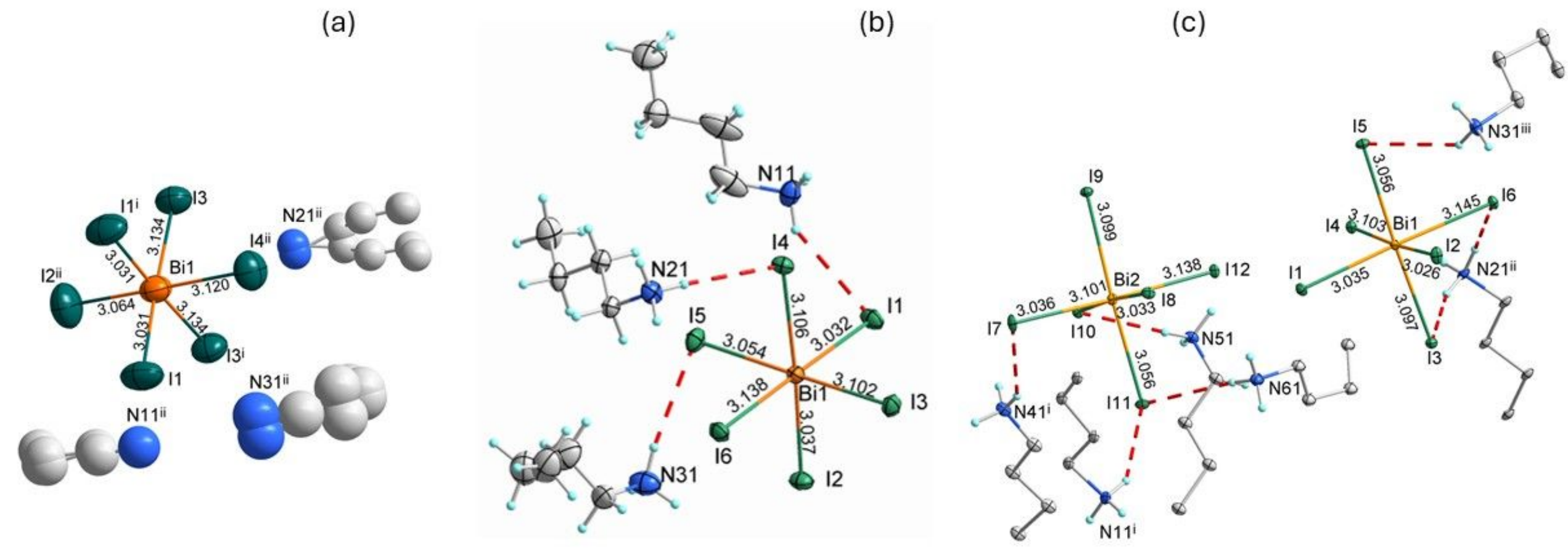

Figure S1 Basic building units of **(But)$_3$[BiI$_6$]** in 295 K (a), 200 K (b), 120 K (c); symmetry codes (295 K – (*i*) *x, y, -z*+1*;* (*ii*) (*x/a, y/b, z/c*)*;* 120 K – (*i*) -0.5+*x*, 0.5-*y,* 1-*z*; (*ii*) 1-*x, -y, z,* (*iii*) 0.5+*x*, 0.5-*y,* 2-*z*).

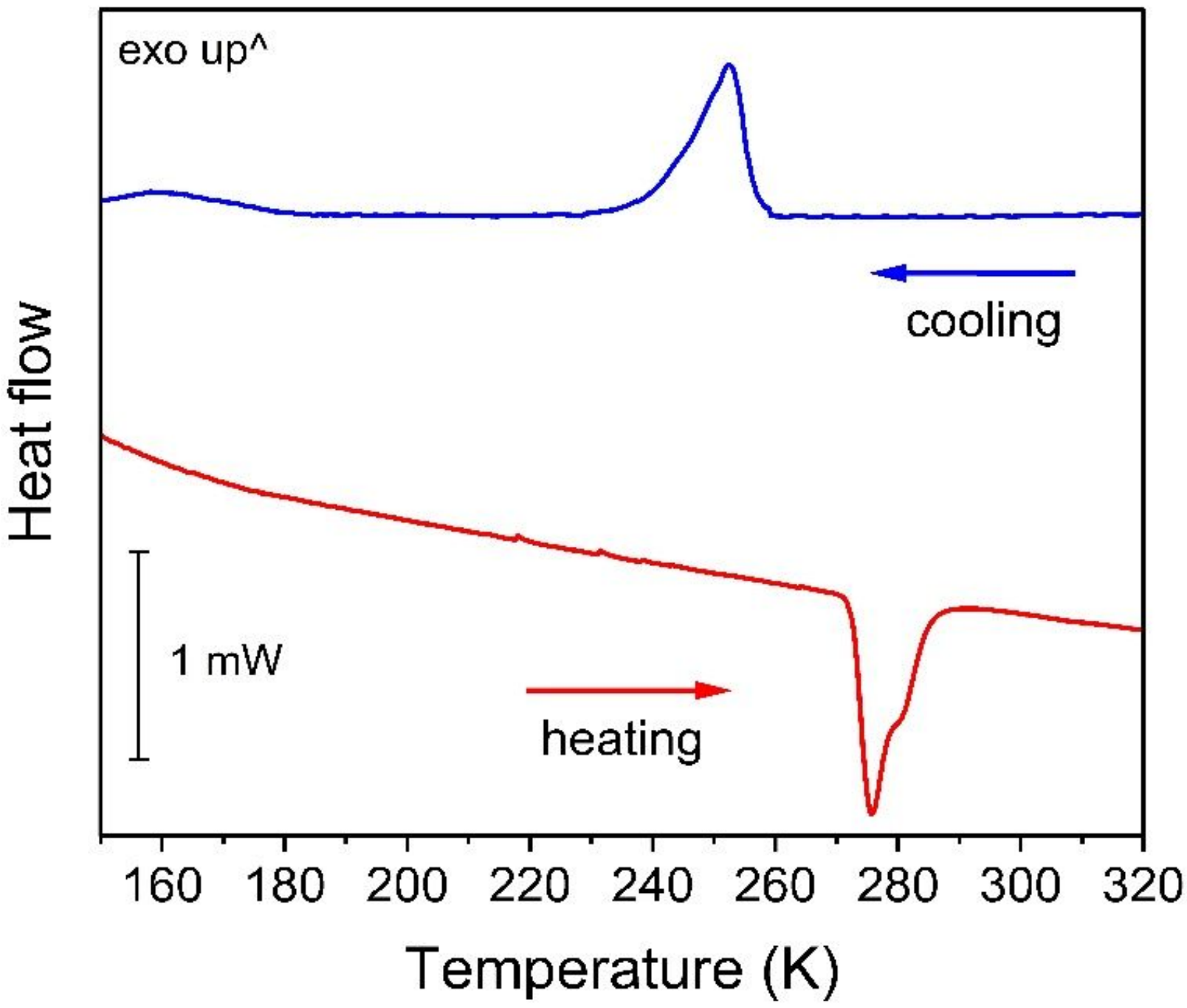

Figure S2 DSC heating and cooling curves for **$(But)_3[BiI_6]$** powder sample showing thermal anomalies associated with the first order I↔II phase transition.

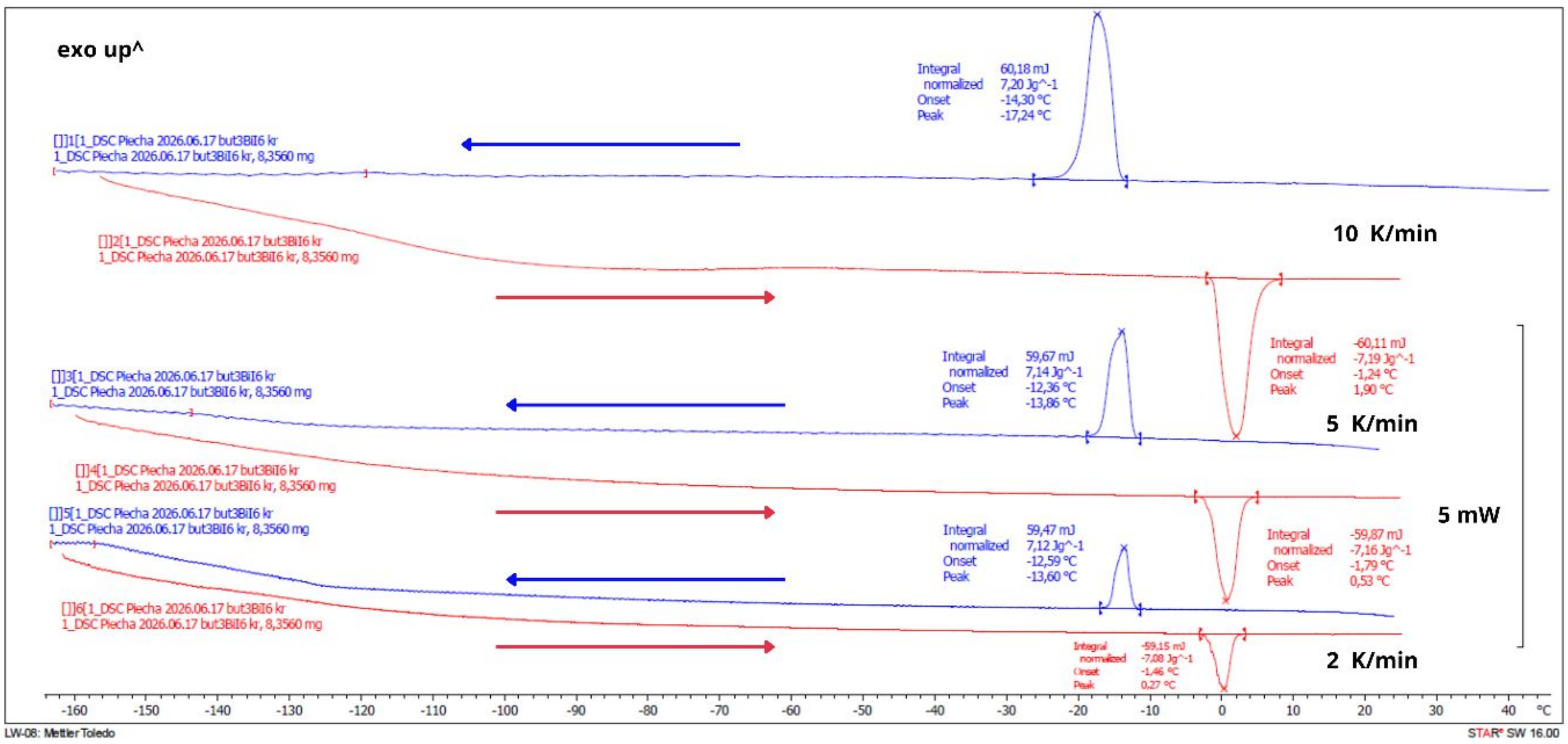

Fig. S3. DSC heating and cooling curves for the $(but)_3[BiI_6]$ single crystal sample (m = 8.3560 mg) measured with different cooling/heating rates.

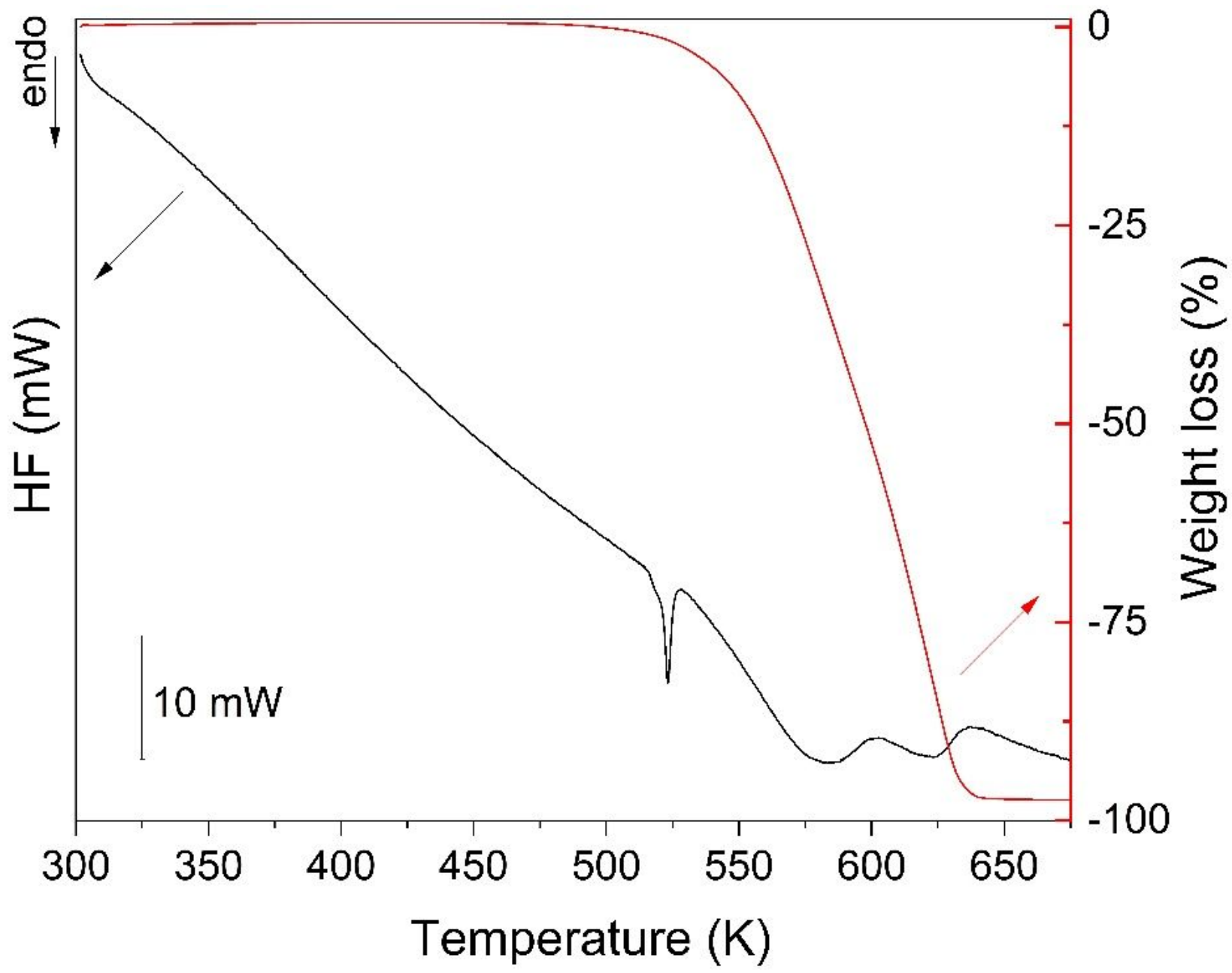

Figure S4 DSC-TGA for **$(But)_3[BiI_6]$** (mass loss in % - red).

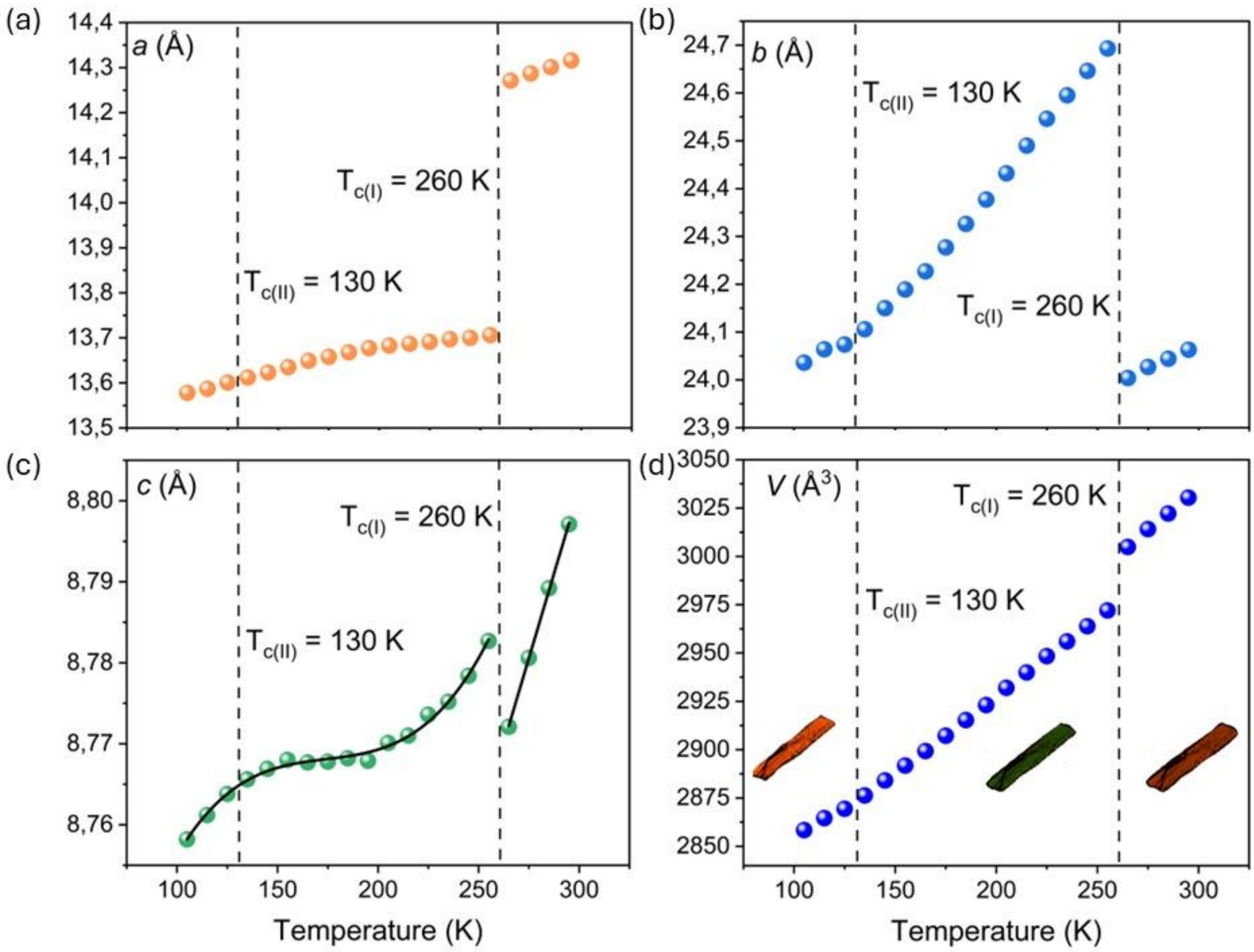

Figure S5 Lattice parameters (a-c) and volume (d) changes in the temperature function; thermochromic behavior of the crystal in different phases (d).

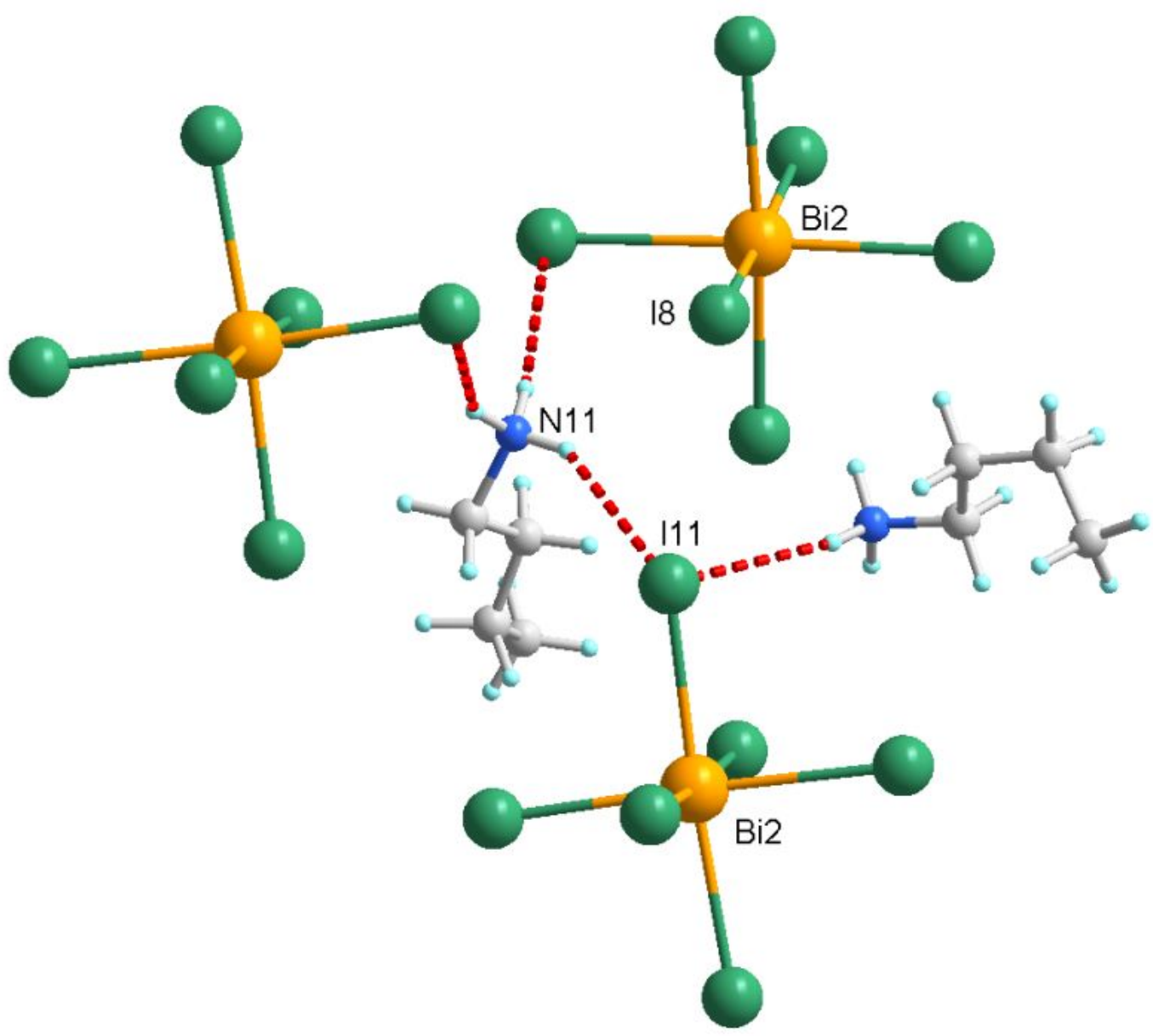

Figure S6 Hydrogen bonds between N11 butylammonium cation and 3 different octahedra with shortest I8-I11 distance visible.

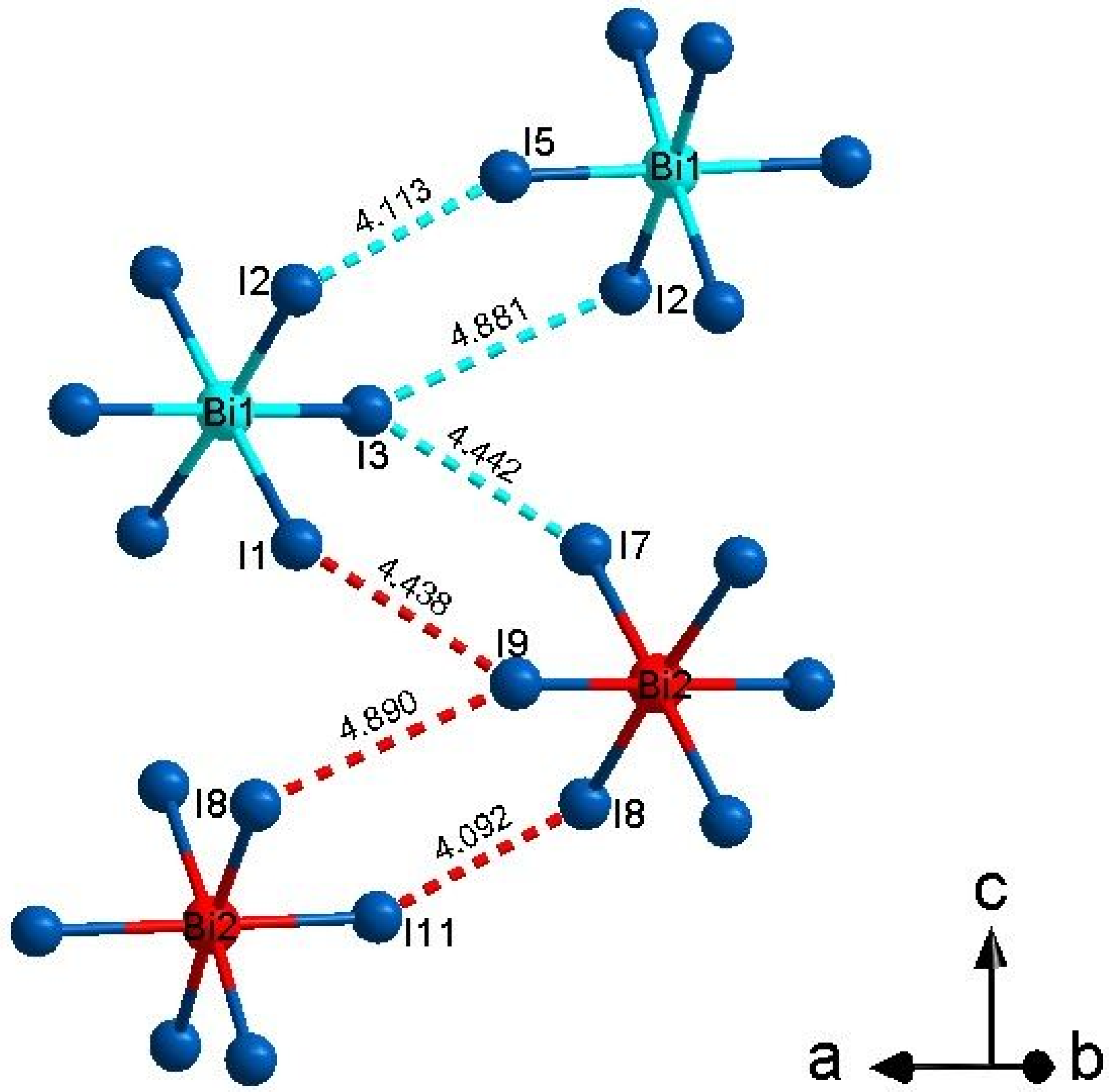

Figure S7 I···I in **$(But)_3[BiI_6]$** distances at 120 K.

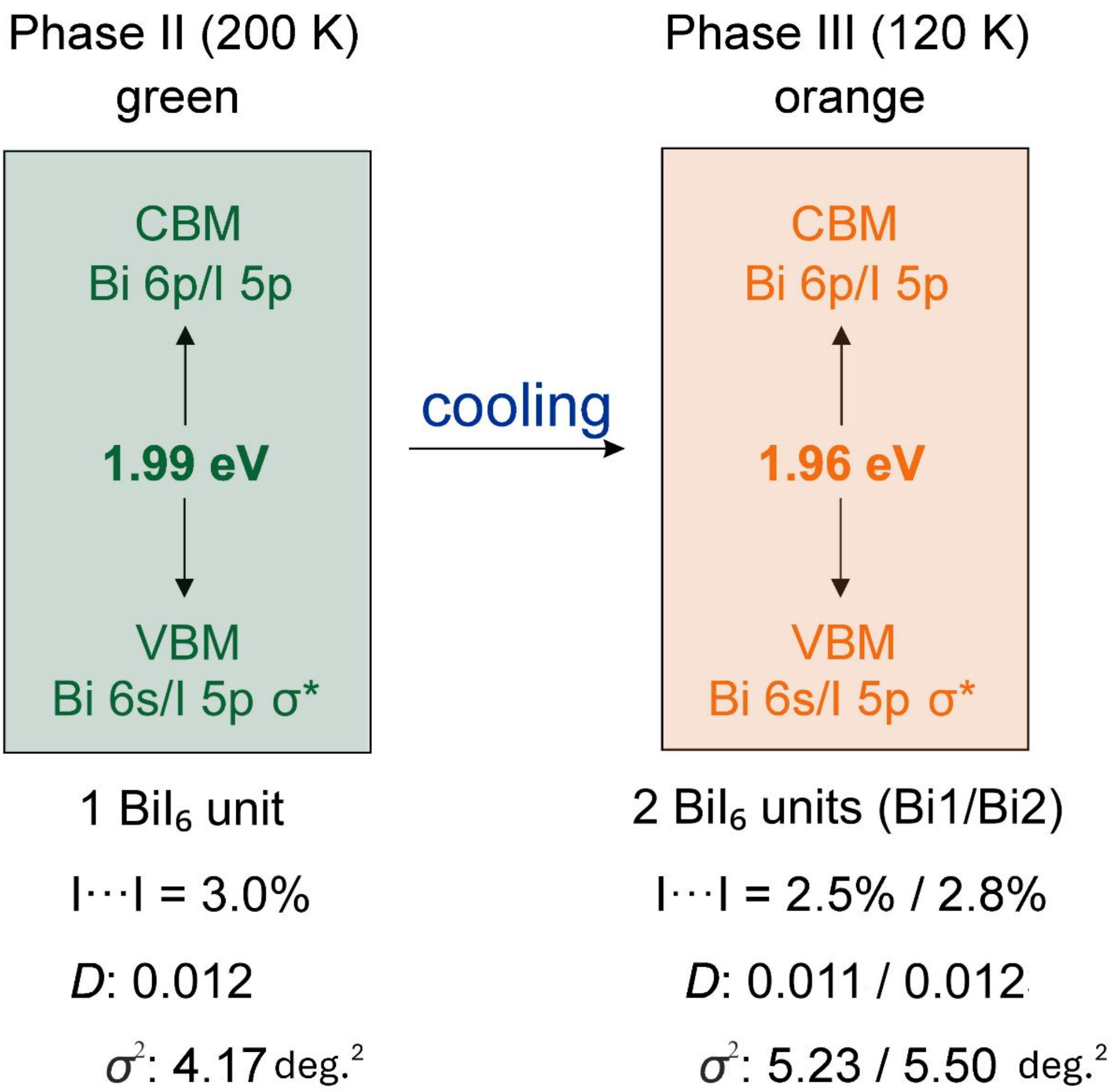

Scheme S1 Correlation between I···I interactions, distortion indexes (*D*), the bond angle variance (σ^2, $(°)^2$), and the resulting electronic transitions in Phase **II** and Phase **III**.

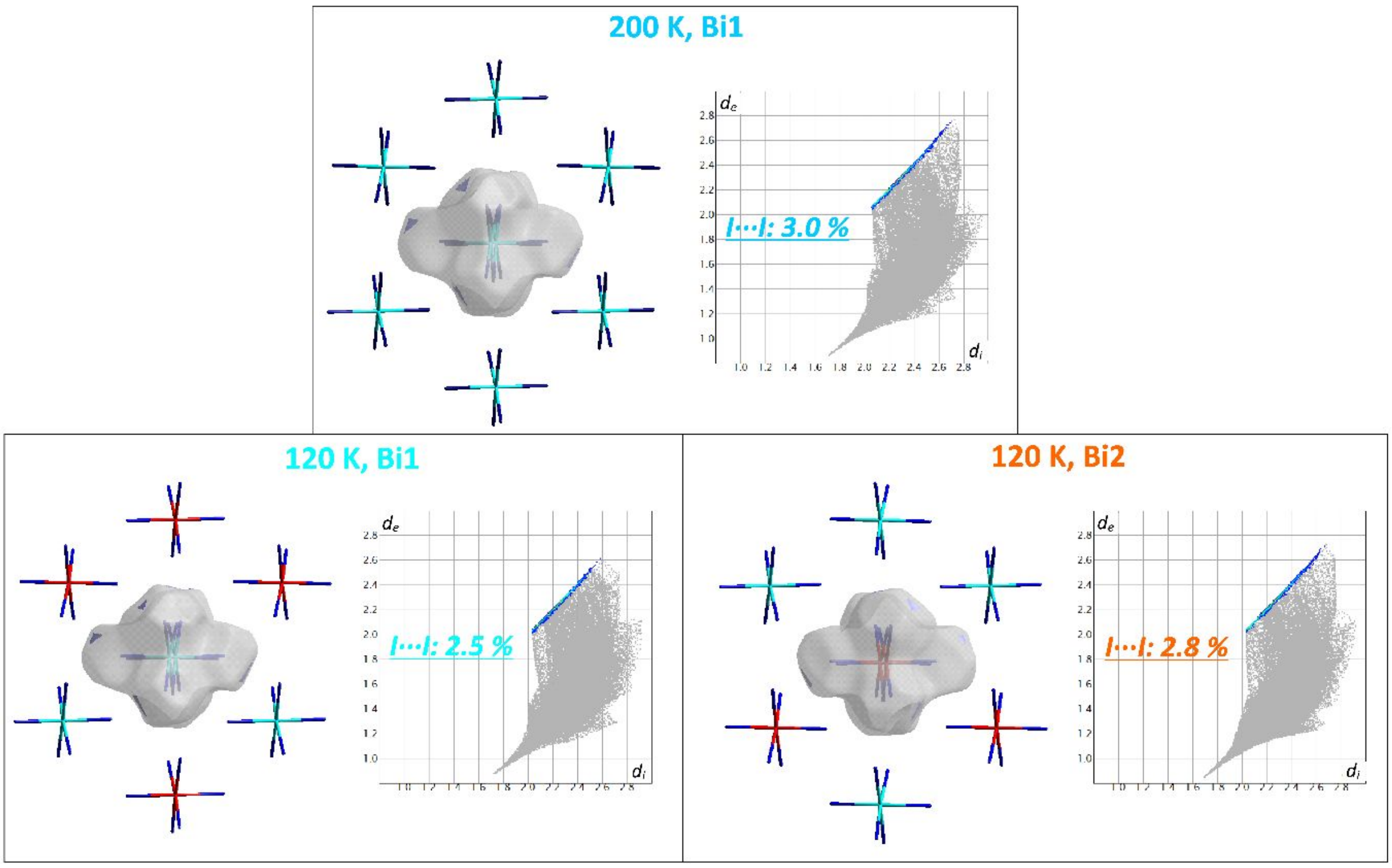

Figure S8 Comparison of I···I contacts and the corresponding fingerprint features in the two phases (200 K and 120 K).

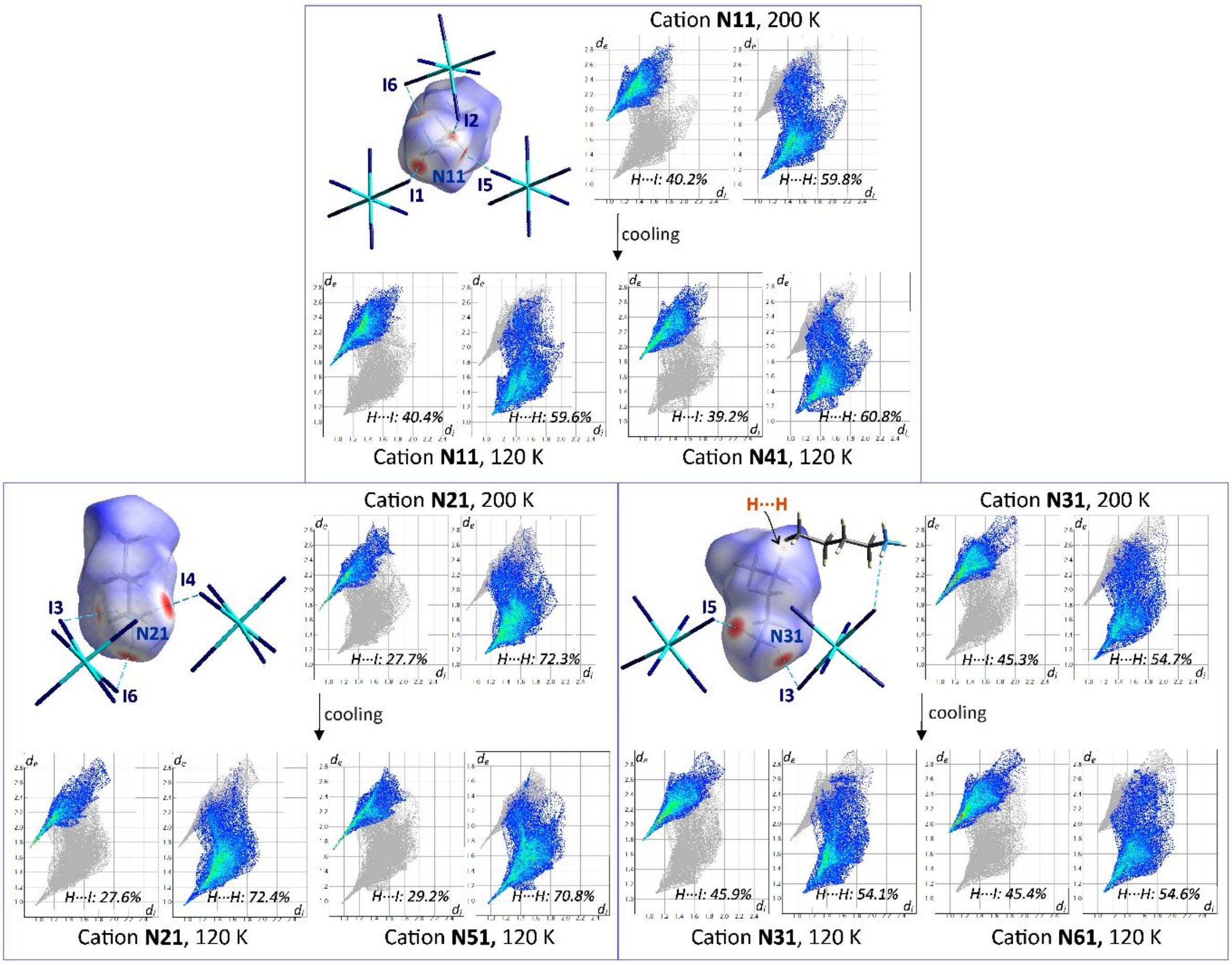

Figure S9 Hirshfeld surfaces of the butylammonium cations N11, N21, and N31 at 200 K mapped over d_{norm}. The corresponding 2D fingerprint plots show H···I and H···H interactions for both 200 K and 120 K; at 120 K, the phase transition results in six symmetry-independent cations (N11/N41, N21/N51, and N31/N61).

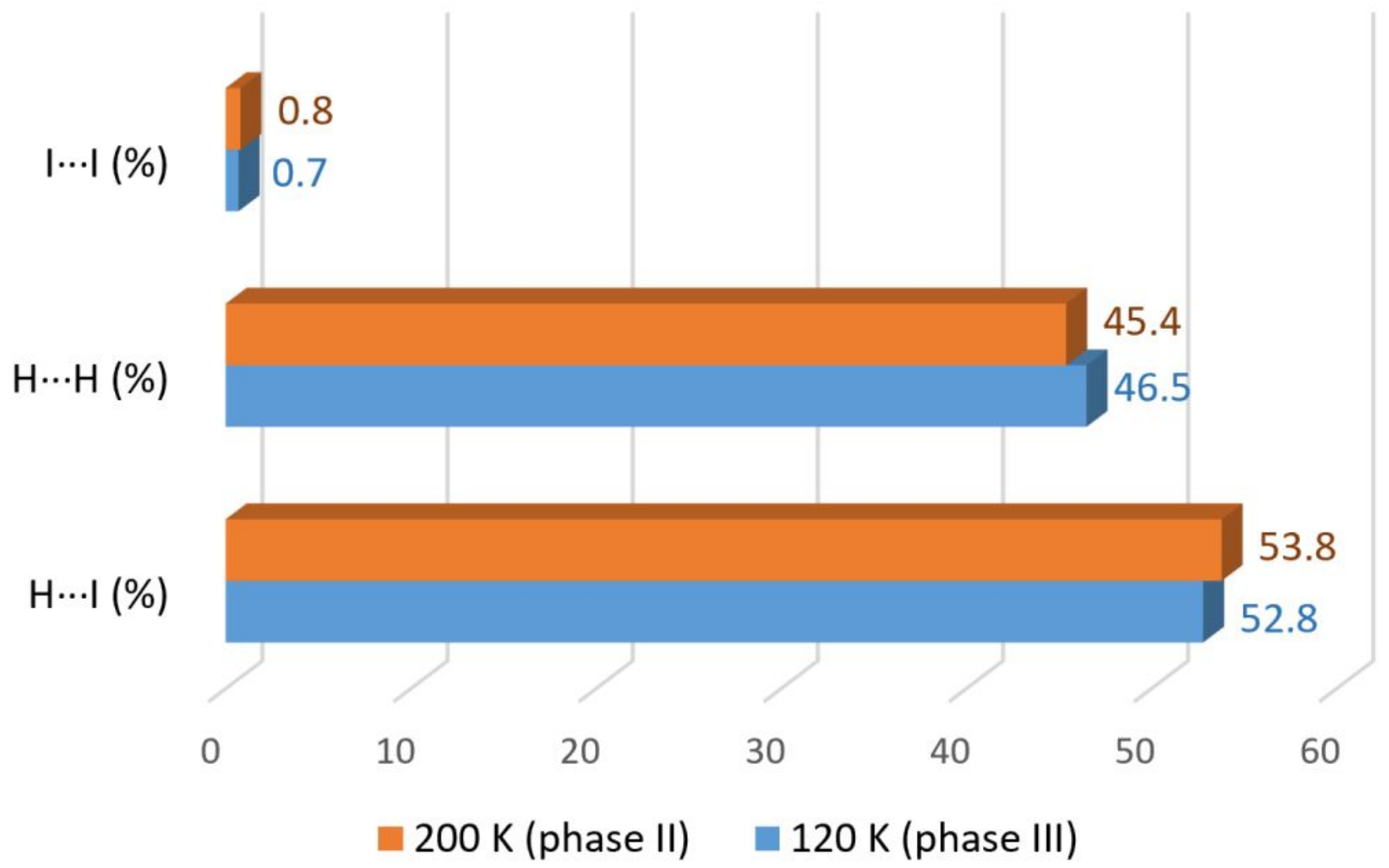

Figure S10 Averaged Hirshfeld surface contact contributions for phase II (200 K) and phase III (120 K), calculated as arithmetic means over all symmetry-independent molecular fragments (three butylammonium cations and one BiI6 octahedron at 200 K; six cations and two octahedra at 120 K).

Powder X-ray diffraction

The PXRD measurements conducted at 295, 200, and 120 K further confirm the presence of three orthorhombic phases in **$(But)_3[BiI_6]$**. At room temperature, the diffraction pattern (Figure S10) shows a (210) reflection consistent with *Pnnm* symmetry; this reflection is systematically absent in both *Pccn* and $P2_12_12$ phases. Additionally, the (110) reflection shifts toward higher 2θ angles upon cooling, reflecting the contraction of lattice parameters.

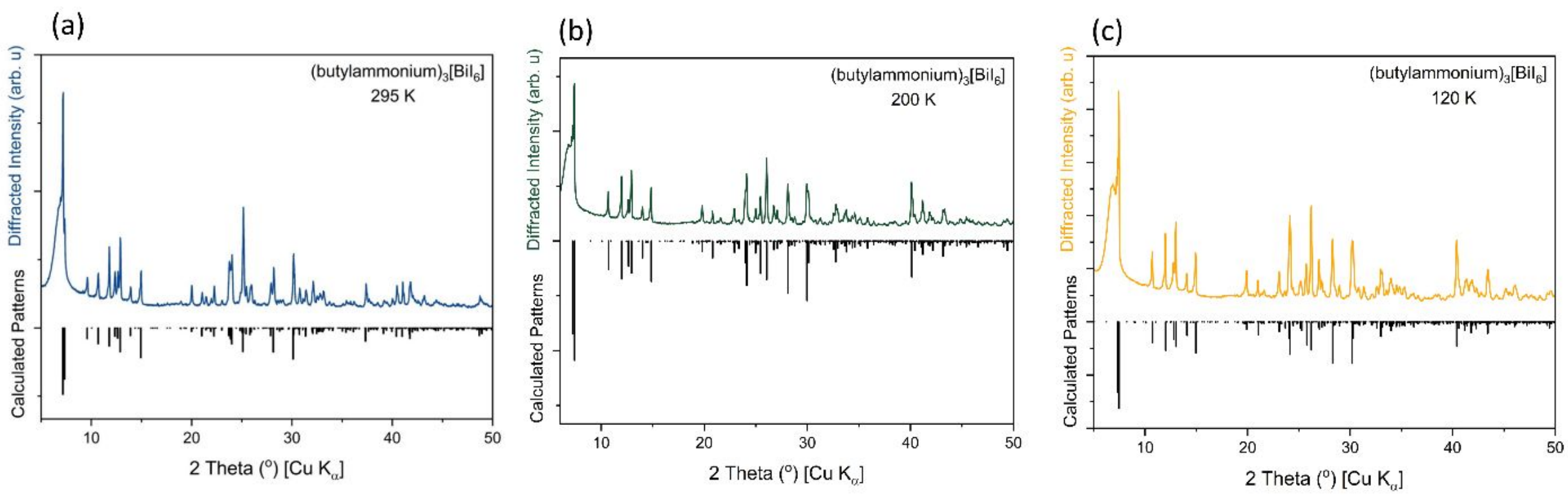

Figure S11 Experimental and calculated diffractogram of **$(But)_3[BiI_6]$** in 295 K (a), 200 K (b), 120 K (c).

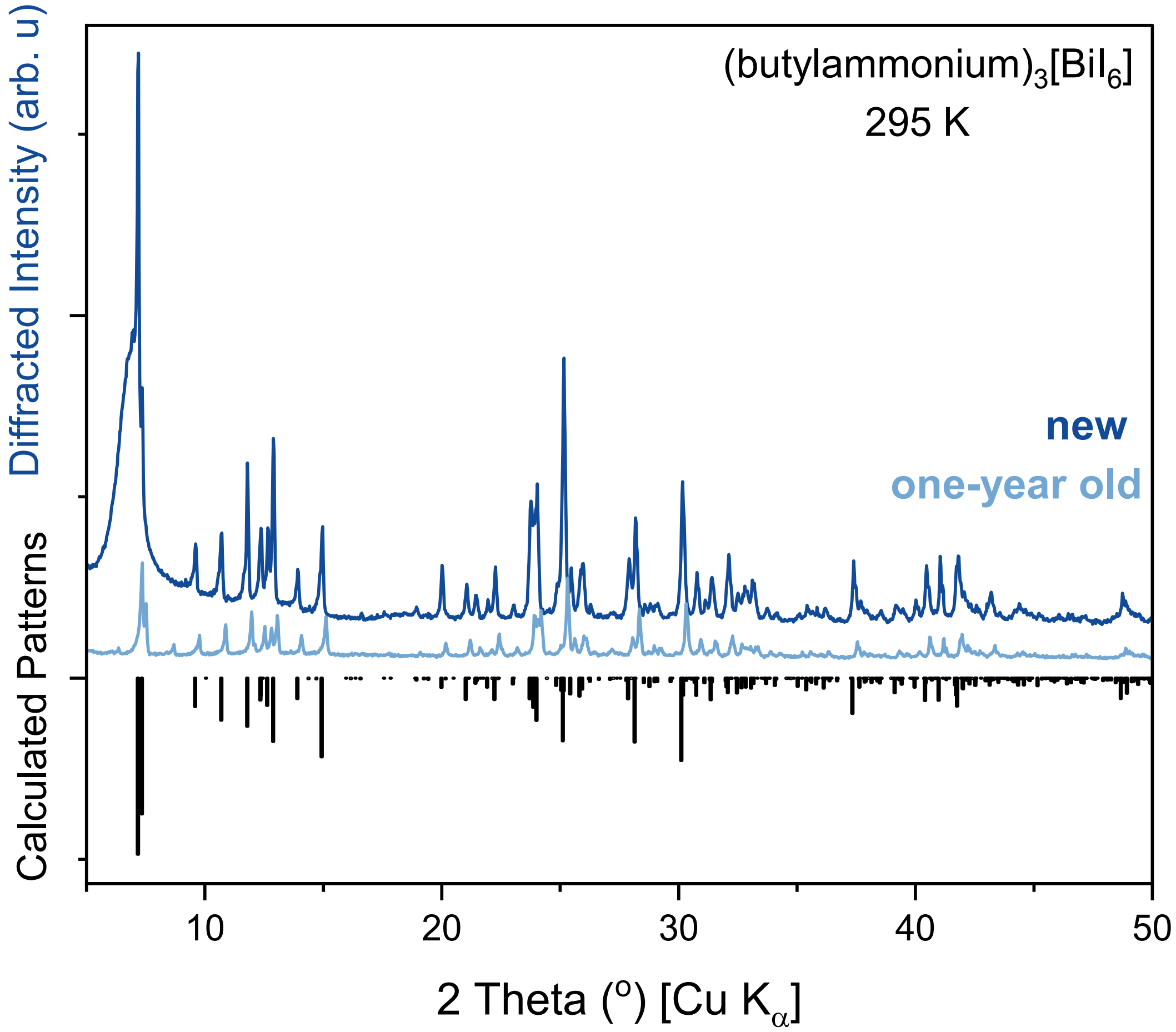

Figure S12 Experimental and calculated diffractogram of **$(But)_3[BiI_6]$** in 295 K for new powder (navy) and for 1-year old powder (light blue).

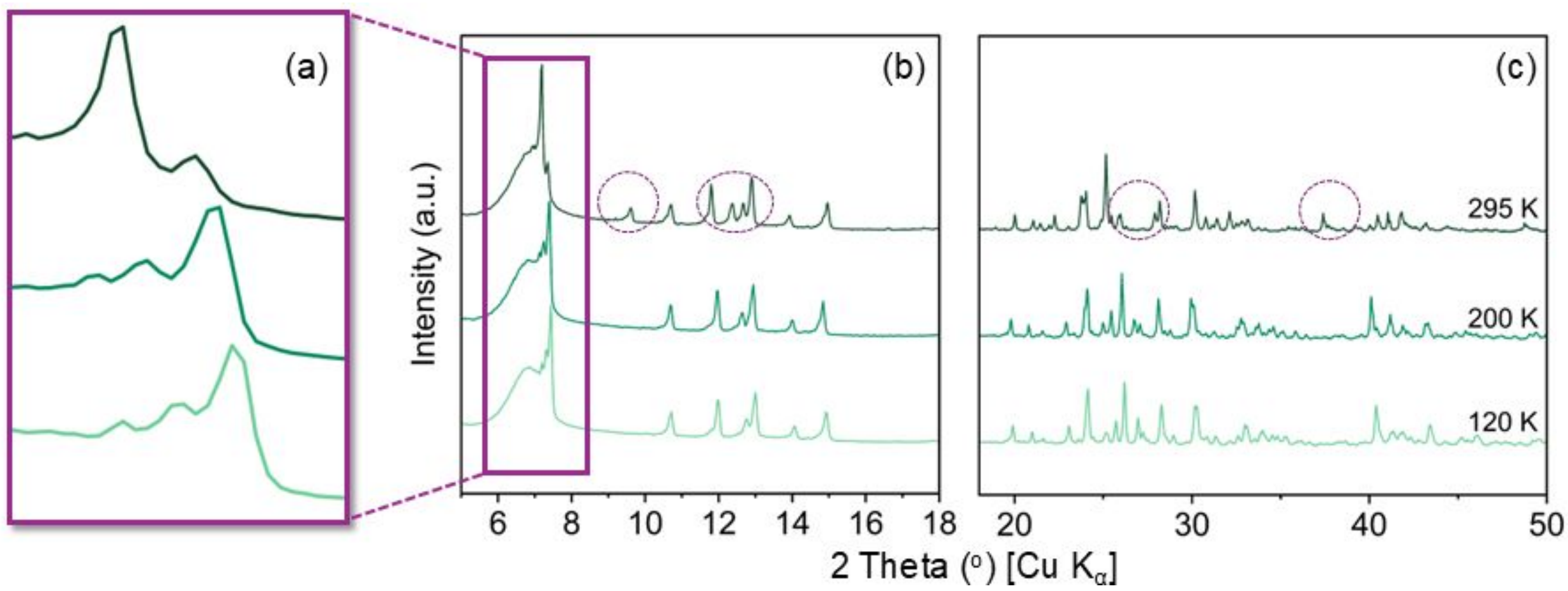

Figure S13 PXRD diffractograms for **$(But)_3[BiI_6]$** in 295, 200 and 120 K respectively; (a) showing the (hkl) reflection shift (b) 5-18 angle range (c) 18-50 angle range.

Second harmonic generation (SHG) experiments were performed using a laser system employing a wavelength-tunable Topaz Prime Vis-NIR optical parametric amplifier (OPA) pumped by Coherent Astrella Ti:Sapphire regenerative amplifier providing femtosecond laser pulses (800 nm, 75 fs) at 1 kHz repetition rate. The output of OPA was set to 1500 nm and the laser fluence at samples was equal 0.20 mJ/cm^2. The single crystals of **$(But)_3[BiI_6]$** and KDP were crushed with a spatula and sieved through an Aldrich mini-sieve set, collecting a microcrystal size fraction of 88–125 μm. Next, size-graded samples were fixed in-between microscope glass slides to form tightly packed layers, sealed, and mounted to the horizontally aligned sample holder. No refractive index matching oil was used. The employed measurement setup operates in the reflection mode. Specifically, the laser beam was directed onto the sample at 45 degrees to its surface. Emission collecting optics consisted of a Ø25.0 mm plano-convex lens of focal length 25.4 mm mounted to the 400 μm 0.22 NA glass optical fibre and was placed along the normal to the sample surface. The distance between collection lens and the sample was equal to 30 mm. The spectra of the temperature-dependent NLO responses were recorded by an Ocean Optics Flame T XR fiber-coupled CCD spectrograph with 200 μm entrance slit. Scattered pumping radiation was suppressed with the use of a Thorlabs 750 nm hard-coated short-pass dielectric filter. The temperature control of the sample was performed using a Linkam LTS420 Heating/Freezing Stage. Temperature stability was equal to 0.1 K. TR-SHG study of **$(But)_3[BiI_6]$** was conducted in a range from 83 K to 333 K. Each individual data point of TR-SHG carries about 10% uncertainty. Three individual samples of **$(But)_3[BiI_6]$** were measured and display the same behavior. Kurtz-Perry powder test was performed by comparing the SHG signal of **$(But)_3[BiI_6]$** at 83K with that of the KDP standard at 293K.

Measurement Conditions

In the case of SHG measurements these were made in airconditioned laser room, stable temperature of 20 deg. C with typical RH of 50%. As is the case with typical laser measurements, measurements were made under dark conditions.

Regarding the dielectric measurements, sample preparation was carried out under ambient laboratory conditions (approximately 23 °C and typical indoor humidity). The experiments were performed in a measurement cell immersed in liquid nitrogen and continuously purged with gaseous nitrogen. The temperature was varied at a rate of 3 K min^{-1}. The dielectric measurements were conducted inside a closed measurement cell, without direct exposure to ambient light. To assess the reproducibility and short-term stability of the dielectric response, selected samples were sealed in glass ampoules after the measurements and remeasured after 2–3 days, yielding comparable dielectric responses.

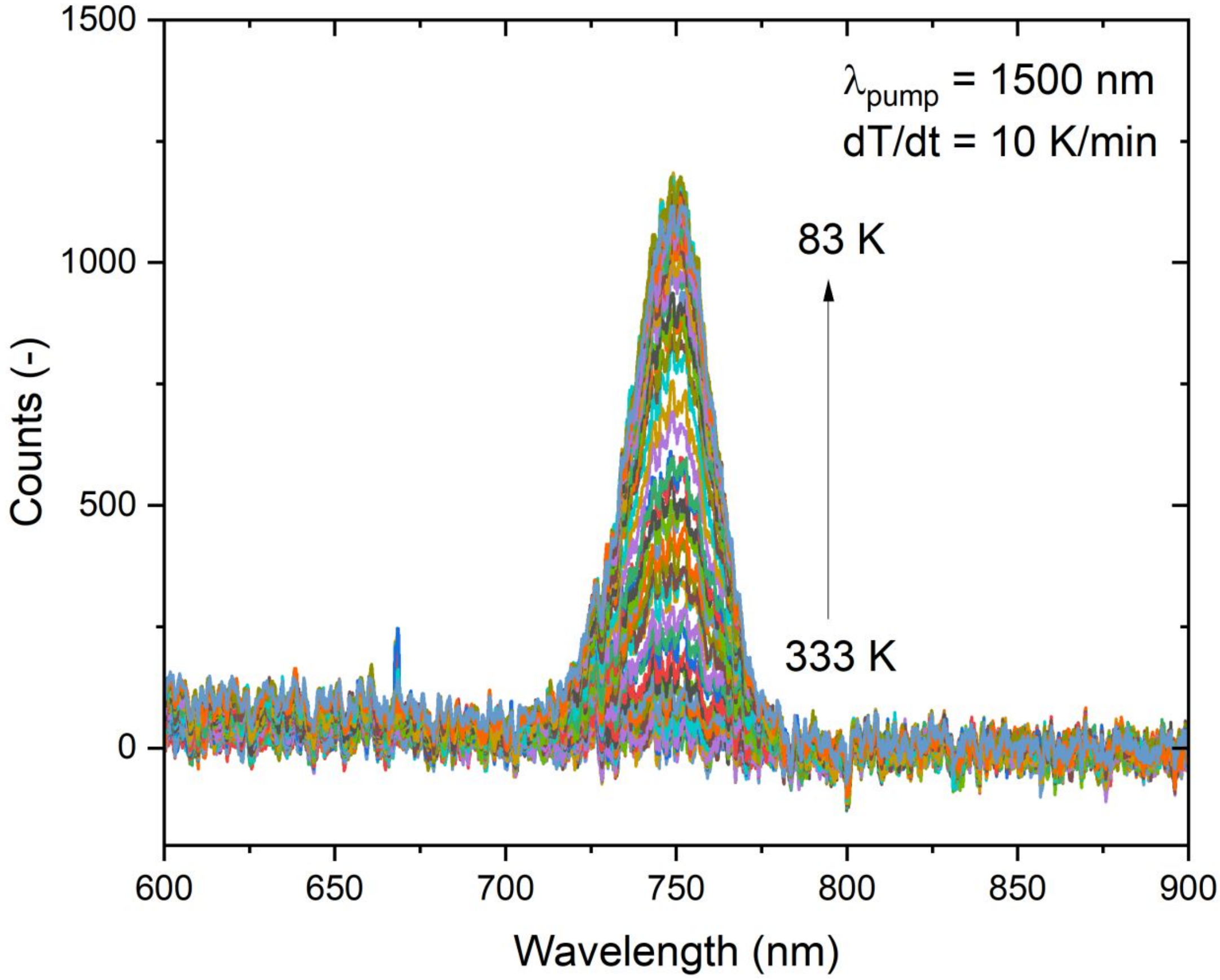

Figure S14 SHG spectra for **$(But)_3[BiI_6]$** collected during a cooling run from 333 K to 83 K.

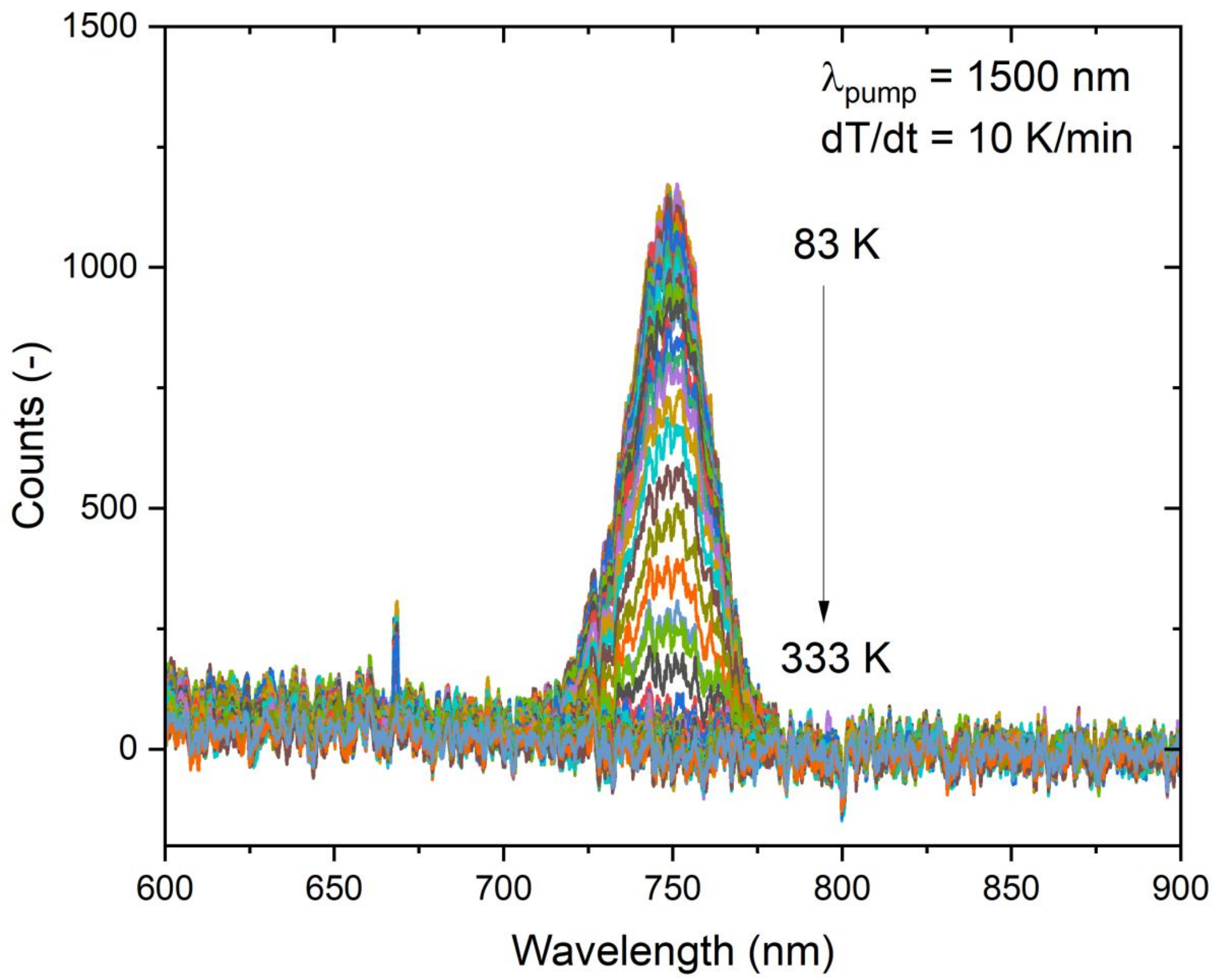

Figure S15 SHG spectra for **$(But)_3[BiI_6]$** collected during a heating run from 83 K to 333 K.

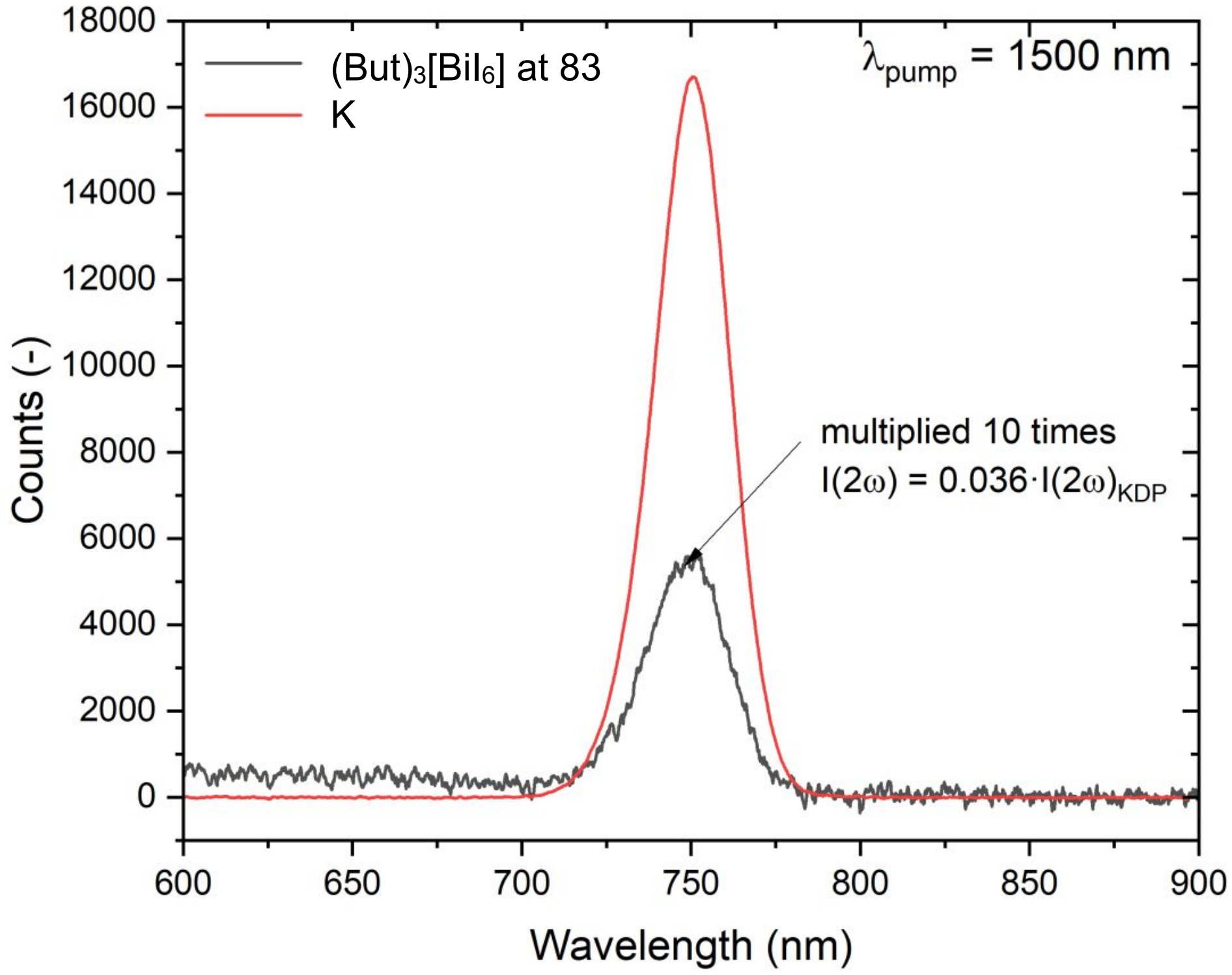

Figure S16 SHG spectra of **$(But)_3[BiI_6]$** at 83 K and KDP at 293 K, acquired using identical excitation and signal collection geometries. The SHG trace has been multiplied by a factor of 10 to improve visibility.

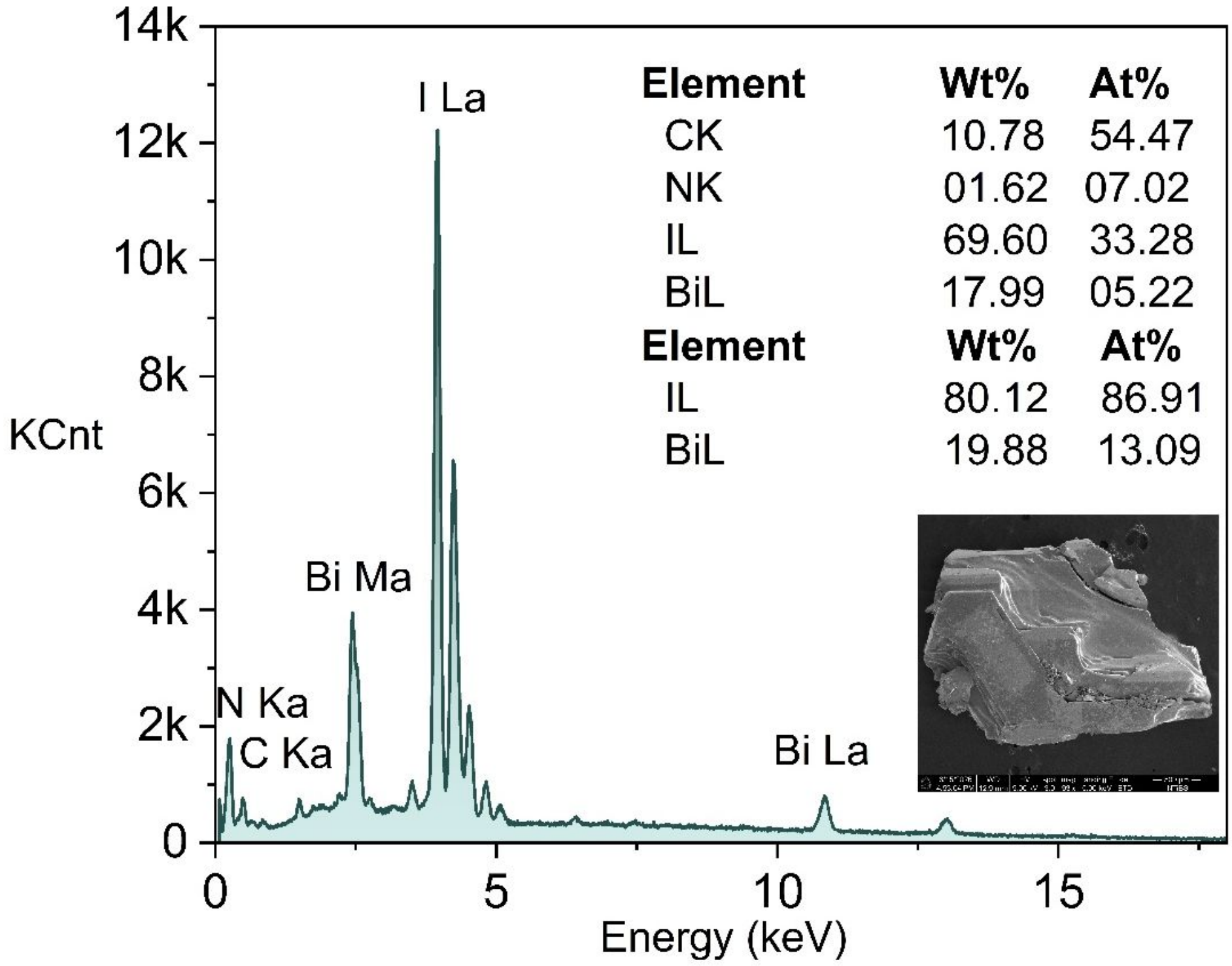

Figure S17 SEM-EDS result of **$(But)_3[BiI_6]$** confirming the composition of obtained crystals.

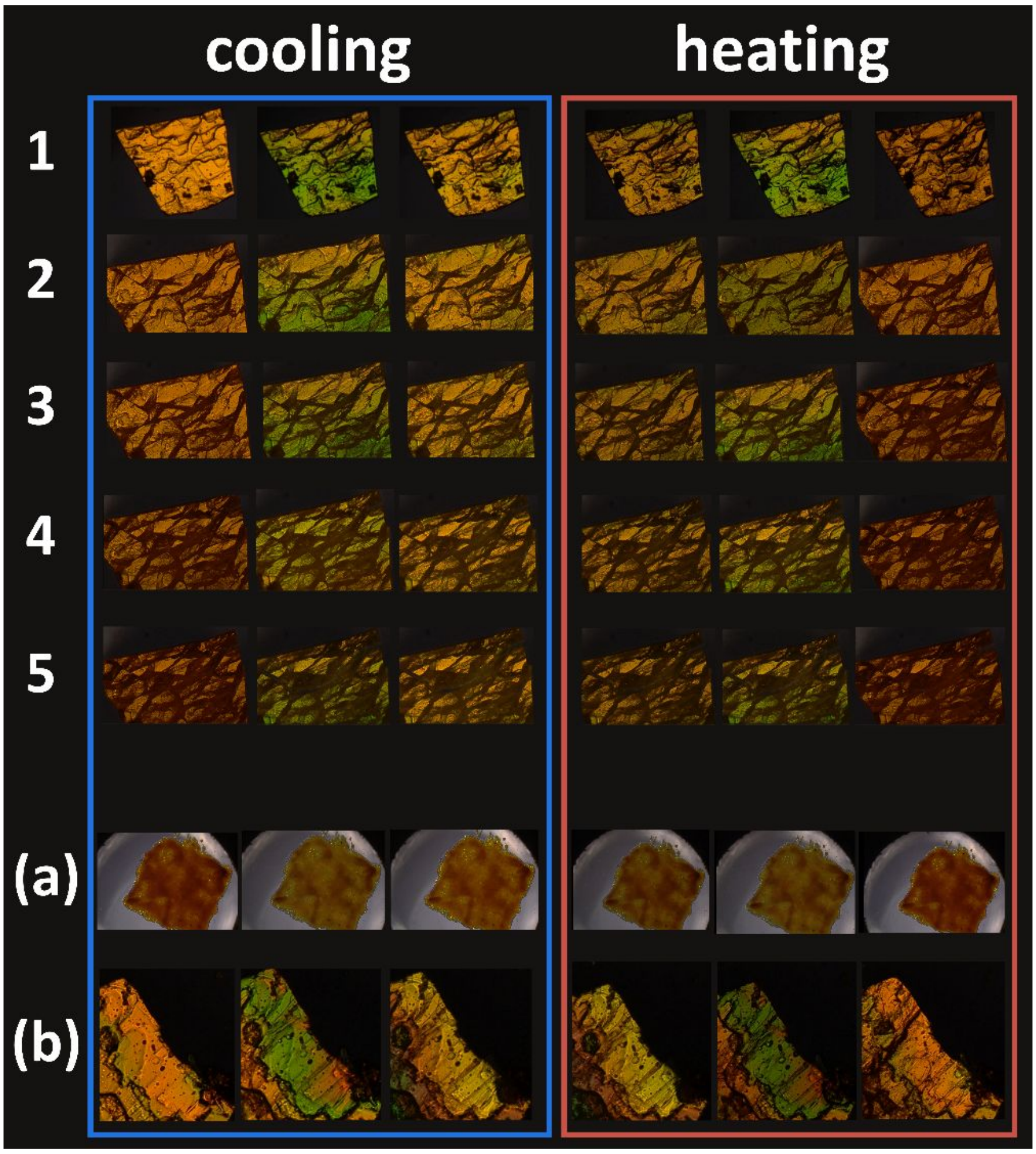

Figure S18 Thermochromic behavior of **$(But)_3[BiI_6]$** for 5 cooling-heating cycles (1-5); for 1-year old powder (a) and for twinned defected crystal (b).
